# ITGB1 Drives Hepatocellular Carcinoma Progression by Modulating Cell Cycle Process Through PXN/YWHAZ/AKT Pathways

**DOI:** 10.3389/fcell.2021.711149

**Published:** 2021-12-17

**Authors:** Jinghe Xie, Tingting Guo, Zhiyong Zhong, Ning Wang, Yan Liang, Weiping Zeng, Shoupei Liu, Qicong Chen, Xianglian Tang, Haibin Wu, Shuai Zhang, Keqiang Ma, Bailin Wang, Yimeng Ou, Weili Gu, Honglin Chen, Yaqi Qiu, Yuyou Duan

**Affiliations:** ^1^ School of Biomedical Sciences and Engineering, Guangzhou International Campus, South China University of Technology, Guangzhou, China; ^2^ Laboratory of Stem Cells and Translational Medicine, Institutes for Life Sciences and School of Medicine, South China University of Technology, Guangzhou, China; ^3^ School of Biology and Biological Engineering, South China University of Technology, Guangzhou, China; ^4^ Department of Gastroenterology and Hepatology, Guangzhou Digestive Disease Center, Guangzhou First People’s Hospital, School of Medicine, South China University of Technology, Guangzhou, China; ^5^ Department of Hepatobiliary Pancreatic Surgery, Huadu District People’s Hospital of Guangzhou, Guangzhou, China; ^6^ Department of General Surgery, Guangzhou Red Cross Hospital, Jinan University, Guangzhou, China; ^7^ Department of General Surgery, the First Affiliated Hospital of Guangdong Pharmaceutical University, Guangzhou, China; ^8^ National Engineering Research Center for Tissue Restoration and Reconstruction, South China University of Technology, Guangzhou, China; ^9^ Key Laboratory of Biomedical Engineering of Guangdong Province, South China University of Technology, Guangzhou, China; ^10^ Key Laboratory of Biomedical Materials and Engineering of the Ministry of Education of China, South China University of Technology, Guangzhou, China; ^11^ Innovation Center for Tissue Restoration and Reconstruction, South China University of Technology, Guangzhou, China

**Keywords:** hepatocellular carcinoma, integrin β1 (ITGB1), cell cycle progression, paxillin (PXN), 14-3-3 protein zeta (YWHAZ), AKT

## Abstract

Integrin β1 (ITGB1), which acts as an extracellular matrix (ECM) receptor, has gained increasing attention as a therapeutic target for the treatment of hepatocellular carcinoma (HCC). However, the underpinning mechanism of how ITGB1 drives HCC progression remains elusive. In this study, we first found that ITGB1 expression was significantly higher in HCC tissues than in normal controls by bioinformatics analysis. Furthermore, bioinformatics analysis revealed that paxillin (PXN) and 14-3-3 protein zeta (YWHAZ) are the molecules participating in ITGB1-regulated HCC tumor cell cycle progression. Indeed, immunohistochemistry (IHC) revealed that ITGB1, paxillin, and YWHAZ were strongly upregulated in paired HCC tissue compared with adjacent normal tissues. Notably, the inhibition of ITGB1 expression by small interfering RNA (siRNA) resulted in the downregulated expression of PXN and YWHAZ in primary HCC cells, as assessed by western blot and immunostaining. In addition, ITGB1 knockdown markedly impaired the aggressive behavior of HCC tumor cells and delayed cell cycle progression as determined by cell migration assay, drug-resistance analysis, colony formation assay, quantitative real-time polymerase chain reaction (qRT-PCR), and cell cycle analysis as well as cell viability measurements. More importantly, we proved that xenograft ITGB1^high^ tumors grew more rapidly than ITGB1^low^ tumors. Altogether, our study showed that the ITGB1/PXN/YWHAZ/protein kinase B (AKT) axis enhances HCC progression by accelerating the cell cycle process, which offers a promising approach to halt HCC tumor growth.

## Introduction

Hepatocellular carcinoma (HCC), as the most prevalent subtype of primary hepatic malignancy, has been ranked as the fourth leading cause of cancer-related mortality worldwide (Bray et al., 2018). In the initial stage, HCC is asymptomatic that always leads to the failure to timely diagnosis (Kim and Viatour, 2020). Indeed, the majority of HCC are not detectable until they reach an advanced stage, rendering HCC patients ineligible for receiving curative therapies (Rasool et al., 2014). Consequently, being one of the most aggressive malignancies, HCC results in very poor clinical outcomes in patients, whose overall 5-year survival rate is estimated to be extremely low (Hu et al., 2019). Hence, the impetus for elucidating the molecular mechanisms governing the tumor progression of HCC is still strongly required, which will be beneficial to identify a novel prognostic biomarker and therapeutic target for HCC.

Integrin β1 (ITGB1), which constitutes the largest subfamily of integrins, is well characterized as a cell surface receptor that participates in a variety of physiological and pathophysiological processes (Hynes, 2002). Moreover, accumulating evidence implicates that ITGB1 is aberrantly expressed in multiple cancer types, including breast cancer, lung cancer, gastric cancer, prostate cancer, pancreatic cancer, colorectal cancer, and laryngeal cancer, and contributes to malignant phenotypes of tumors by mediating cell migration, invasion, survival, and apoptosis (Bogorad et al., 2014; Li et al., 2018; Sun et al., 2018).

In the context of HCC progression, profound extracellular matrix (ECM) remodeling has been detected in the tumor microenvironment of HCC (Zhang et al., 2020a). Therefore, ITGB1, which functions as an ECM receptor, might be a promising target for inhibiting HCC growth. Indeed, knockdown of ITGB1 expression effectively halted HCC progression in *in vivo* HCC mouse model (Bogorad et al., 2014). Nevertheless, the spontaneous HCC mouse model employed in the study was driven by overexpression of human MET/ΔN90-β-catenin oncogenes, which do not faithfully replicate human HCC initiation and progression. Furthermore, the authors focused solely on the investigation of ITGB1 in regulating epithelial–mesenchymal transition (EMT) and its associated signaling pathways. It has also been acknowledged that ITGB1 plays a critical role in promoting EMT process of HCC through activating downstream effector focal adhesion kinase (FAK) (Guo et al., 2020). However, the majority of published studies investigating the role of ITGB1 in HCC were performed on HCC cell lines, which might not accurately reflect genotypic and phenotypic features of the original tumors (Gillet et al., 2013; Jiang et al., 2015). Additionally, the underpinning mechanism of how the aberrant expression of ITGB1 confers HCC cells the sustained proliferative capacity still remains largely unexplored.

In this study, we better clarified the oncogenic role and crucial molecular mechanism of ITGB1 on HCC progression by utilizing the liver hepatocellular carcinoma (LIHC) dataset retrieved from The Cancer Genome Atlas (TCGA) and functional enrichment analysis. Next, we evaluated the expression of ITGB1 in the tumor specimens of HCC patients. Then, the predicted function and mechanisms of ITGB1 were verified in primary tumor cells derived from tissue specimens of three HCC patients as well as in primary tumor cell-derived xenograft model. Overall, our study demonstrates that ITGB1 exerts a pivotal role in accelerating cell cycle *via* paxillin (PXN)/14-3-3 protein zeta (YWHAZ)/protein kinase B (AKT) pathways, which provides a novel insight into the role of ITGB1 in HCC malignant progression.

## Materials and Methods

### The Cancer Genome Atlas Database

The data of LIHC (*n* = 369) and LIHC adjacent normal samples (*n* = 50) were obtained from The Cancer Genome Atlas (TCGA) website. We also downloaded the clinicopathological parameters of LIHC patients from TCGA for correlation analysis.

### Clinical Specimens

Ten LIHC tissues and matched non-tumor tissues were collected from LIHC patients, who underwent surgery at Guangzhou First People’s Hospital of South China University of Technology. The methodologies used in the present study were approved by the ethics committee at Guangzhou First People’s Hospital and were conducted in accordance with the Declaration of Helsinki.

### Isolation and Culture of Primary Hepatocellular Carcinoma Cells From Liver Hepatocellular Carcinoma Tissues

Fresh tumor tissues obtained from LIHC patients were minced into 1-mm^3^ pieces and digested in a mixture of DMEM (Thermo Fisher, United States), type IV collagenase (Sigma, United Kingdom), DNase I, and penicillin–streptomycin for 1 h. Then, we used a 40-μm cell strainer (BD Biosciences, United States) to filter disaggregated cell suspension. After lysis of red blood cells, the remaining cells were washed with DMEM for three times and resuspended in DMEM supplemented with 10% FBS. We seeded these cells onto dishes and incubated them in a 5% CO_2_ incubator at 37°C. The medium was refreshed twice a week. When the cells reached 70%–80% confluency, they were passaged serially until passage 10.

### Methylation-Specific Polymerase Chain Reaction

Genomic DNA of 10 LIHC tissues and paired non-tumor tissues was extracted according to manufacturer’s instructions by using a TIANamp Genomic DNA kit (Tiangen Biotech, China). We then used Methylation-Gold Kit (Zymo, United States) for the bisulfite treatment according to manufacturer’s instructions. Next, the website http://www.urogene.org/cgi-bin/methprimer/methprimer.cgi was used to analyze the CpG map of the ITGB1 promoter and the location of primers. One CpG island was found in the sequence of ITGB1 promoter region. The primers for the methylated ITGB1 CpG island were 5′-ACG​GTT​TCG​ATT​TGT​TTC​GGG​ATT​TA-3′ and 5′-CCG​TAT​TCT​TTA​AAT​TCC​TCT​TCC​TCG -3′. The primers for the unmethylated ITGB1 CpG island were 5′-ATG​GTT​TTG​ATT​TGT​TTT​GGG​ATT​TA-3′ and 5′-CCA​TAT​TCT​TTA​AAT​TCC​TCT​TCC​TCA -3′. Finally, PCR products were separated by using agarose gel electrophoresis.

### Immunohistochemistry Staining

Tumor tissues and matched non-tumor tissues derived from 10 LIHC patients were incubated in 4% paraformaldehyde overnight, followed by embedding in paraffin. The tissues were sectioned at a thickness of 4 μm. Then, the tissues were processed by deparaffinization, rehydration, and antigen retrieval. The sections were blocked in a commercial goat serum (Boster Biological Technology, United States) and incubated with the following antibodies: ITGB1 (1:1,000; Abcam, United States), PXN (1:1,000; Abcam, United States), and YWHAZ (1:1,000; Absin, China) at 4°C overnight. The next day, we used SP Rabbit and Mouse HRP Kit (CWBIO, China) to amplify signal. The sections were then counter-stained with hematoxylin, dehydrated with a graded ethanol series, and cleared with xylene. Images were acquired by Aperio CS2 Digital Pathology Slide Scanners (Leica, Germany). The analysis of IHC staining signal was performed by ImageJ.

### Cell Transfection Assay

Small interfering RNA (siRNA) against ITGB1 were synthesized by Hanheng (Shanghai, China). The sequences of siRNA for negative control and ITGB1 are listed in Supplementary Table S1. When the cell density reached 50%–70%, transient transfection was performed according to manufacturer’s instructions by importing siRNA into cells.

### Wound-Healing Assay

When HCC cells seeded in 24-well plates reached 80% confluence, we used a 20-μl sterile pipette tip to scratch the surface of the plates for making wound lines. Each well was washed with PBS, and the cells were transfected with ITGB1-specific siRNA or negative control siRNA. The cells were photographed at 0 and 24 h using a light microscope (Nikon, Japan).

### Colony Formation Assay

HCC cells were seeded in six-well plates at a density of 10^3^ cells/well and incubated at 37°C overnight. Then, the cells were transfected with negative control siRNA, ITGB1-specific siRNA, ITGB1-specific siRNA plus PXN (Raybiotech, United States, 1 μg/well), ITGB10-specific siRNA plus YWHAZ (Abcam, United States, 1 μg/well), and ITGB1-specific siRNA plus PXN plus YWHAZ. After 6 days, cells were fixed with 100% methanol, followed by staining with 0.1% violet. Finally, the number of cell colonies was counted using a light microscope (Nikon, Japan). We considered a cluster that contains more than 50 cells as a colony.

### Cell Viability Analysis

PrestoBlue™ HS Cell Viability Reagent (Invitrogen, United States) was used to perform cell viability analysis according to manufacturer’s instructions. Briefly, HCC cells were transfected with ITGB1-specific siRNA or negative control siRNA for 24, 48, and 72 h, followed by incubation in PrestoBlue™ HS at 37°C for 10 min. Then, Cytation5 Imaging Plate Reader (BioTek, United States) was utilized to assess cell viability.

### Cell Cycle Analysis

Cell cycle distribution was determined by a cell cycle and apoptosis analysis kit (Meilunbio, Dalian, China) according to manufacturer’s protocols. In brief, HCC cells were transfected with ITGB1-specific siRNA or negative control siRNA for 48 h. Then, these cells were fixed with 70% cold ethanol at 4°C overnight. The next day, the cells were washed with cold PBS and stained with the mixture of propidium iodide and RNase A. The cell cycle was detected by flow cytometry (BD Biosciences, United States).

### Quantitative Real-Time Polymerase Chain Reaction

Total RNA of LIHC tissues and paired non-tumor tissues or HCC cells treated with negative control siRNA, ITGB1-specific siRNA, ITGB1-specific siRNA plus PXN, ITGB1-specific siRNA plus YWHAZ, and ITGB1-specific siRNA plus PXN plus YWHAZ was isolated using the Universal RNA Extraction Kit (TaKaRa, Japan) according to manufacturer’s instructions. Then, the total RNA of tissues or cells was reverse-transcribed to cDNA by 5X PrimeScript RT Master Mix (TaKaRa, Japan). PowerUp™ SYBR™ Green Master Mix (Applied Biosystems, United States) and QuantStudio™ 1 Real-Time PCR instrument (Applied Biosystems, United States) were applied to quantitatively evaluate the levels of targeted gene expression. GAPDH was set as the internal control. QuantStudio™ Design and Analysis software v1.5.1 (Applied Biosystems, United States) was employed to analyze raw data and define relative quantity (RQ). The sequences of the primers are listed in Supplementary Table S2.

### Western Blot

HCC cells were collected after being transfected with ITGB1-specific siRNA or negative control siRNA for 72 h. Then, these cells were lysed in the RIPA buffer (Solarbio, Beijing, China). For each sample, 70 μg protein was separated by SDS-PAGE, followed by transferring to PVDF membranes. Then, the membranes were incubated with the following antibodies: ITGB1 (1:1,000; Abcam, United States), PXN (1:1,000; Abcam, United States), YWHAZ (1:1,000; Absin, China), and AKT (1:1,000; Cell Signaling Technology, United States) at 4°C overnight. The next day, we incubated the membranes with HRP-conjugated secondary antibody (1:1,000; Cell Signaling Technology, United States) at room temperature for 1 h. The ECL detection kit (Affinity Biosciences, China) was employed to examine the specific proteins. We used GAPDH (1:1,000; Cell Signaling Technology, United States) as loading controls.

### Differential Expression Analysis

A differential gene expression analysis was performed between LIHC patients and normal samples by utilizing the DESeq2 Bioconductor package. The gene expression was quantified according to read count. Up- and downregulated differentially expressed genes were filtered with a false discovery rate less than 0.05 and |log2FC| more than 1.5.

### Functional Enrichment Analysis

Functional enrichment analysis of differentially expressed genes was conducted by using TBtools. A *p* value less than 0.05 was set as the threshold to identify signal pathways, which were significantly enriched in differentially expressed genes.

### Construction of Protein–Protein Interaction Networks

We used STRING database to analyze protein–protein interaction (PPI) networks of ITGB1 and constructed the network by Cytoscape version 3.8.0. The proteins, whose combined score of the interaction with ITGB1 was more than 0.8, were screened for further analysis.

### Isolation of Integrin β1^high^ and Integrin β1^low^ Subpopulations From Hepatocellular Carcinoma Cells by FACS

HCC cells suspended in PBS containing 1% bovine serum albumin were stained with mouse anti-human ITGB1 Ab conjugated with PE-Cytm5 (BD Biosciences, United States) or PE-Cytm5 Mouse IgG1 κ Isotype Control (BD Biosciences, United States) at 4°C for 30 min in the dark. The subpopulation of ITGB1^high^ and ITGB1^low^ was obtained using FACSAria SORP cell sorter (BD Biosciences, United States).

### Tumor Mouse Model

ITGB1^high^ and ITGB1^low^ subpopulations isolated from HCC cells (10^6^ cells/mouse) were subcutaneously inoculated into 4-week-old female NOD scid gamma (NSG) mice (BIOCYTOGEN, Nanjing, China; *n* = 5 per group). After 1 month, mice were sacrificed, and tumors were collected for further analysis. We calculated the tumor volume according to the following formula: (length × width^2^) / 2. All animal experiments were carried out with the approval of the Ethical Committee of the South China University of Technology in compliance with Chinese laws and policies.

### Immunofluorescence Analysis

HCC cells derived from LIHC tumor tissues were first seeded in glass-bottom culture dishes (NEST, United States) at 37°C overnight. The next day, HCC cells were transfected with ITGB1-specific siRNA or negative control siRNA. After 72 h, the cells were collected for staining. ITGB1^high^ and ITGB1^low^ HCC tumors formed in mice were collected and embedded in OCT, and the frozen tissues were sectioned at 5 µm. All samples were fixed in 4% paraformaldehyde, permeabilized with 0.5% Triton-X, and blocked in a commercial goat serum (Boster Biological Technology, United States). Then, the samples were stained with AFP (1:100; R and D system, Australia), asialoglycoprotein receptor cyclin (ASGPR) (1:100, Santa Cruz, United States), ITGB1 (1:100; Abcam, United States), ITGB1 (1:100; Cell Signaling Technology, United States), PXN (1:100; Abcam, United States), YWHAZ (1:100; Absin, China), AKT (1:100; Cell Signaling Technology, United States), and Ki67 (1:100; Cell Signaling Technology, United States) at 4°C overnight. We used secondary antibodies for staining at room temperature 1 h: anti-rabbit Alexa 594 (1:1,000, Cell Signaling Technology, United States), anti-rabbit Alexa 488 (1:1,000, Cell Signaling Technology, United States), anti-mouse Alexa 594 (1:1,000, Cell Signaling Technology, United States), and anti-mouse Alexa 488 (1:1,000, Cell Signaling Technology, United States). Cell nuclei were counterstained with DAPI (Beyotime Biotechnology, China). Nikon Ti-E A1 confocal laser-scanning microscope (Nikon, Japan) was used to capture immunostaining images.

We utilized ImageJ to measure the fluorescence of immunostaining signals and the colocalization area of the red and green channels. The values of immunostaining signals were normalized to the total cells visualized by DAPI.

### Statistical Analysis

Statistical analysis between the two groups was performed by Student’s *t*-test using GraphPad 8.0 software (La Jolla, California, United States). The correlation between ITGB1 expression and clinicopathological characteristics was assessed by *χ* ^2^ test. Wilcoxon rank test was used to analyze the gene methylation level of ITGB1 between normal samples and LIHC. Log-rank test was applied to determine *p* values of the Kaplan–Meier curves of overall survival. The threshold used to categorize the patients in low and high ITGB1 is median except ITGB1, because when the threshold is the median, the overall survival between the two groups is not significant. Pearson product-moment correlation coefficient was employed to evaluate the association between two variables. All of the above statistical analyses were conducted by R version 4.0.2. A *p* value <0.05 was considered statistically significant.

## Results

### Integrin β1 Was Highly Expressed in Human Liver Hepatocellular Carcinoma

**FIGURE 1:**
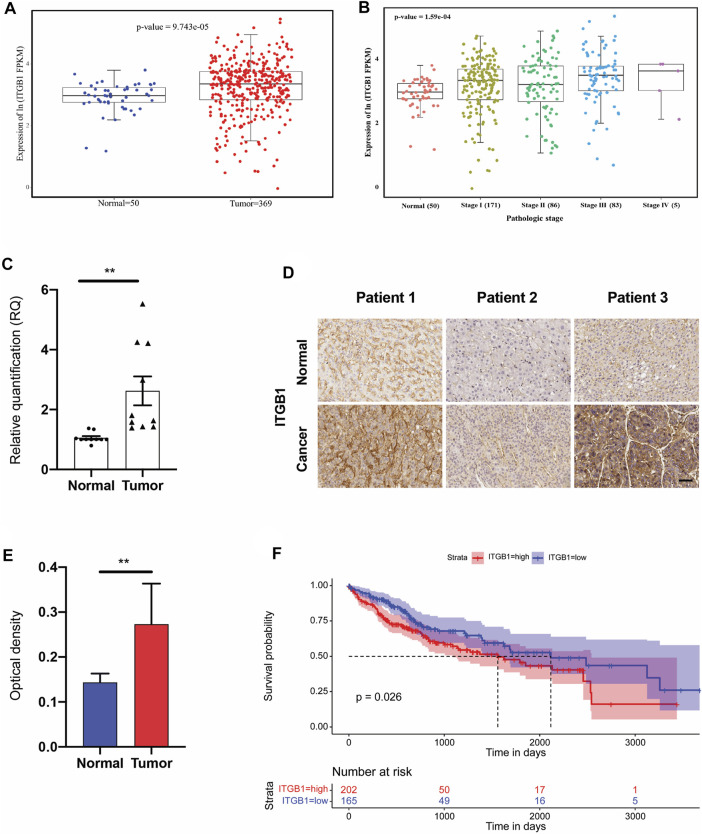
ITGB1 was highly expressed in human LIHC. **(A)** mRNA expression of ITGB1 in normal tissues (*n* = 50) and LIHC tissues (*n* = 369) derived from TCGA data. **(B)** mRNA expression of ITGB1 in LIHC patients of stage I to IV derived from TCGA data. **(C)** ITGB1 mRNA level of LIHC tissues (*n* =10) was quantified by qRT-PCR and normalized to that of tumor-adjacent normal tissues (*n* = 10). Wilcoxon test was used to perform a statistical test. *p < 0.05, ***p < 0.001, ****p < 0.0001. **(D)** Representative images of ITGB1 immunohistochemical staining in paired tumor-adjacent normal tissues (*n* = 10) and LIHC tissues (*n* = 10). Scale bar = 50 µm. **(E)** ITGB1 protein level of LIHC tissues (*n* = 10) was quantified by immunohistochemical staining and normalized to that of tumor-adjacent normal tissues (*n* = 10). **(F)** Kaplan–Meier overall survival curve was shown according to high and low expression of ITGB1 in LIHC patients derived from TCGA data. Values are presented in mean ± SD. **p < 0.01.

Seeking to perform more clinically relevant assessment of the role of ITGB1 during HCC progression, we first evaluated its transcriptome expression in 369 HCC tissues in comparison with 50 normal tissues by analyzing TCGA dataset. The findings indicated that ITGB1, which was markedly increased in HCC tumors compared to normal tissues, was also progressively upregulated along with clinical stages of HCC patients from I to IV (Figure 1A, B). Next, to further validate these results, we examined ITGB1 expression at RNA level by means of quantitative real-time polymerase chain reaction (qRT-PCR) analysis. In agreement with TCGA data, we observed a prominent increase in ITGB1 expression in HCC tumor tissues as compared to the normal peritumoral tissues (Figure 1C). Additionally, IHC staining further confirmed that HCC tumor tissues displayed remarkably upregulated ITGB1 protein levels (Figure 1D, E). Given the strongly positive ITGB1 expression detected in HCC tumor, we next explored whether accumulated ITGB1 influence the overall survival of HCC patients by dividing the HCC cohort (*n* = 367) into high- and low-expression groups. As expected, patients with low levels of ITGB1 had a remarkably longer survival time than those with high levels (Figure 1F). Furthermore, we analyzed the clinicopathological parameters between the ITGB1-high and -low expression groups and found that ITGB1 up-modulation was significantly correlated with high histological stages (Supplementary Table S1). Of note, ITGB1 demonstrated evidently enhanced expression in females compared to males (Supplementary Table S1). Taken together, these data suggested that ITGB1 could serve as a prognostic marker for HCC.

### Integrin β1 Expression Was Negatively Correlated With Gene Methylation

**FIGURE 2:**
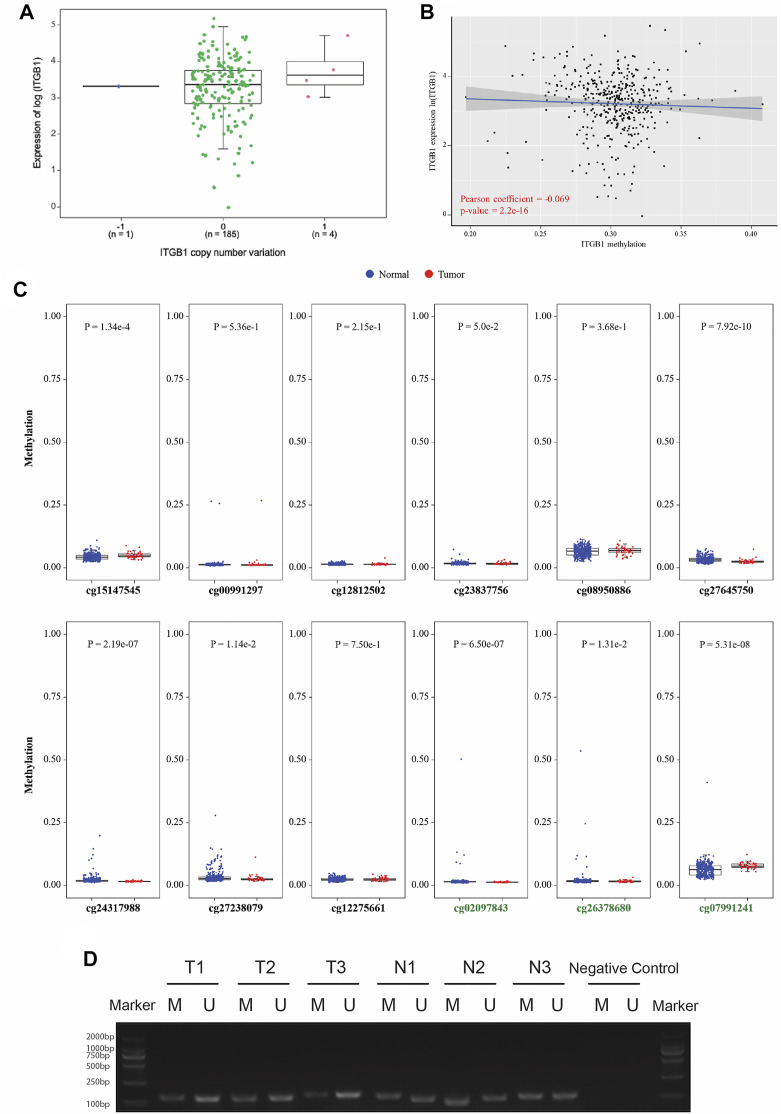
ITGB1 expression was negatively correlated with gene methylation. **(A)** The correlation analysis between ITGB1 expression and gene copy number derived from TCGA data. The *X*-axis presents the gene copy number of ITGB1, and the *Y*-axis presents the gene expression level of ITGB1 quantified as log2 (fragments per kilo-base per million). *p < 0.05, ***p < 0.001, ****p < 0.0001. **(B)** The correlation analysis between ITGB1 expression and DNA methylation derived from TCGA data. The *X*-axis presents the DNA methylation level of ITGB1, and the *Y*-axis presents the gene expression level of ITGB1 quantified as log2 (fragments per kilo-base per million). Pearson correlation coefficient is calculated using cor function in R. **(C)** The methylation levels of CpG islands between HCC and normal hepatocyte samples derived from TCGA data. The *X*-axis presents the methylation sites in CpG islands, and the *Y*-axis presents the degree of methylation (from 0 to 1). Wilcoxon test was used to perform a statistical test. *p < 0.05, ***p < 0.001, ****p < 0.0001. **(D)** Representative image of methylation-specific PCR products for ITGB1 gene in paired tumor-adjacent normal tissues (*n* = 3) and LIHC tissues (*n* = 3). M, methylated sequence; U, unmethylated sequence; T, tumor; N, tumor-adjacent normal tissues.

Motivated by prior results implicating that ITGB1 expression was notably increased in HCC tumor tissues, we sought to elucidate the mechanism underlying the overexpression of ITGB1. It is extensively acknowledged that gene copy number alterations and methylation at different genomic regions are both crucial mechanisms activating oncogene expression (Zhang et al., 2020b). First, we analyzed the gene level focal segment copy number from 190 HCC cases in TCGA dataset. However, no significant alterations of gene copy number were detected for ITGB1 expression in HCC tissues (Figure 2A). Interestingly, we found that the DNA methylation level of ITGB1 was negatively associated with its gene expression in HCC tissues on TCGA database (Figure 2B). Methylation of CpG islands has been demonstrated to play an important role in controlling gene expression through transcriptional inactivation of the corresponding gene (Teodoridis et al., 2004). Therefore, we examined the methylation levels of 12 CpG sites, among which nine CpG sites were within ITGB1 promoter region (black names) and three sites were within ITGB1 5′-untraslated region (green names), between HCC and normal samples (Figure 2C). Remarkably, we observed that, in HCC tumor tissues, five CpG sites within the sequence of ITGB1 promoter region, including cg24317988, cg23837756, cg27238079, and cg27645750, had significantly lower methylation levels than in normal tissues, while higher methylation level was only detected at cg15147545 (Figure 2C). To further verify whether DNA methylation was responsible for ITGB1 overexpression, we evaluated the methylation status of ITGB1 in 10 paired HCC tumor and corresponding non-malignant tissues using methylation-specific PCR (MSP). Results indicated that the methylation level of ITGB1 was decreased in 60% of HCC tumor tissues in contrast with tumor-adjacent normal tissues (Figure 2D and Supplementary Figure S1). Hence, DNA methylation might function as a major mechanism of the aberrant ITGB1 expression in HCC.

### Integrin β1 Inhibition Attenuated the Aggressiveness of Hepatocellular Carcinoma Cells

**FIGURE 3:**
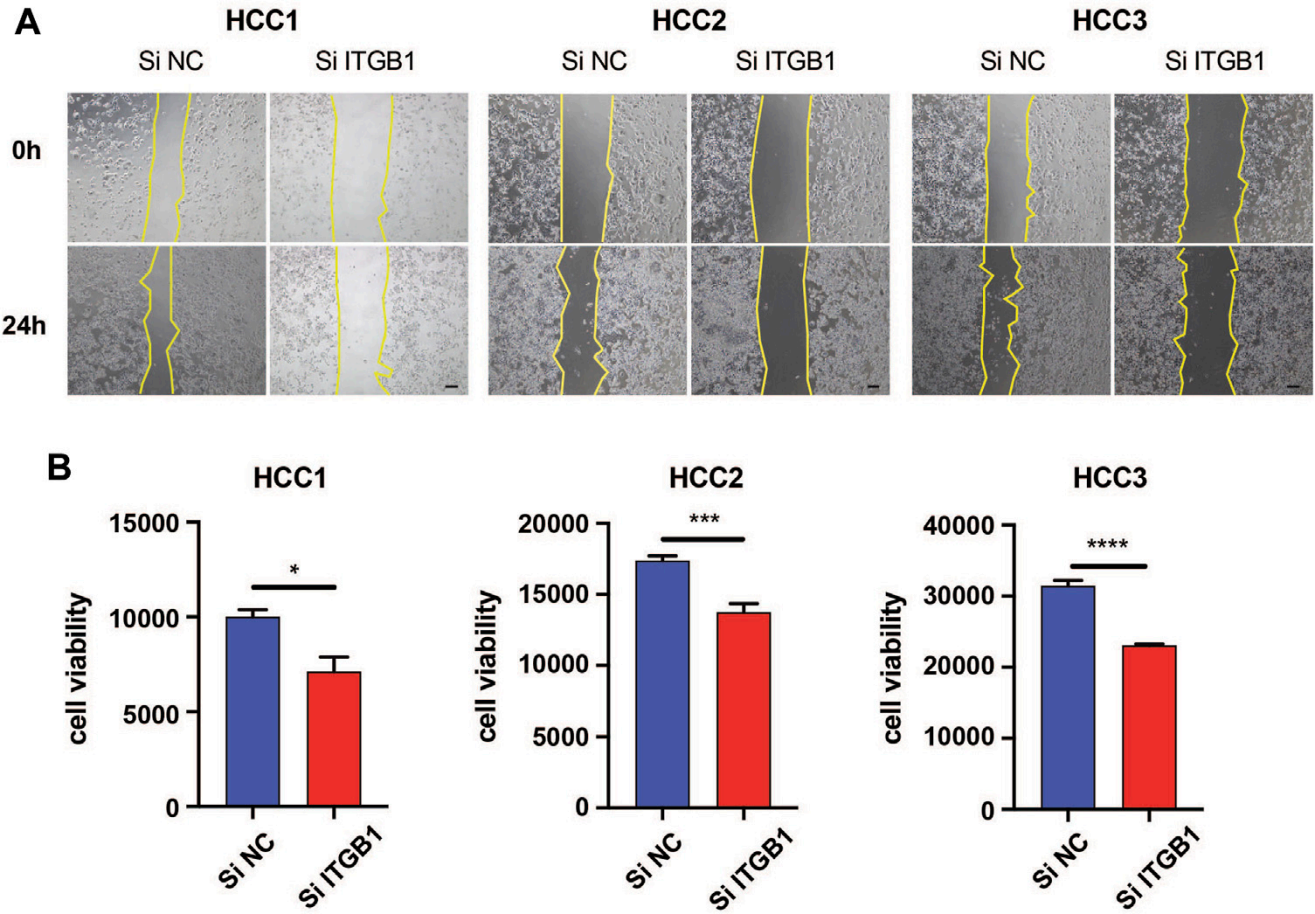
ITGB1 inhibition attenuated the aggressiveness of HCC cells. **(A)** Wound-healing assays were performed to measure cell migration of HCC1, 2, and 3 cells transfected with ITGB1 siRNA for 24 h. Scale bar = 100 µm. **(B)** The effects of ITGB1 siRNA on cell viability of sorafenib-treated HCC1, 2, and 3 cells were measured after 48 h. Values are presented in mean ± SD. *p < 0.05, ***p < 0.001, ****p < 0.0001.

Given that upregulated ITGB1 expression was associated with poor prognosis in HCC, we were wondering whether the inhibition of ITGB1 could impair the aggressiveness of HCC cells. To this end, we first isolated cells from three fresh HCC tumor tissues and confirmed that these cells were primary HCC cells by performing immunostaining of AFP and ASGPR markers (Supplementary Figure S2). Next, we conducted wound-healing assay to ascertain the influence of ITGB1 expression on HCC cell migration. Remarkably, the suppression of ITGB1 by using SiRNA for 24 h hampered HCC cell migration (Figure 3A). In addition, to evaluate if ITGB1 also played a role in regulating drug resistance of HCC cells, we treated HCC cells with siITGB1 and sorafenib for 48 h. Interestingly, the knockdown of ITGB1 profoundly enhanced the antitumor effects of sorafenib (Figure 3B), suggesting that a high level of ITGB1 expression led to reduced sensitivity of HCC cells to antitumor drugs. Collectively, all these evidences revealed that ITGB1 exerted an oncogenic role in accelerating the malignant process of HCC.

### Integrin β1 Promoted the Progression of Hepatocellular Carcinoma by Facilitating Mitotic Cell Cycle

**FIGURE 4:**
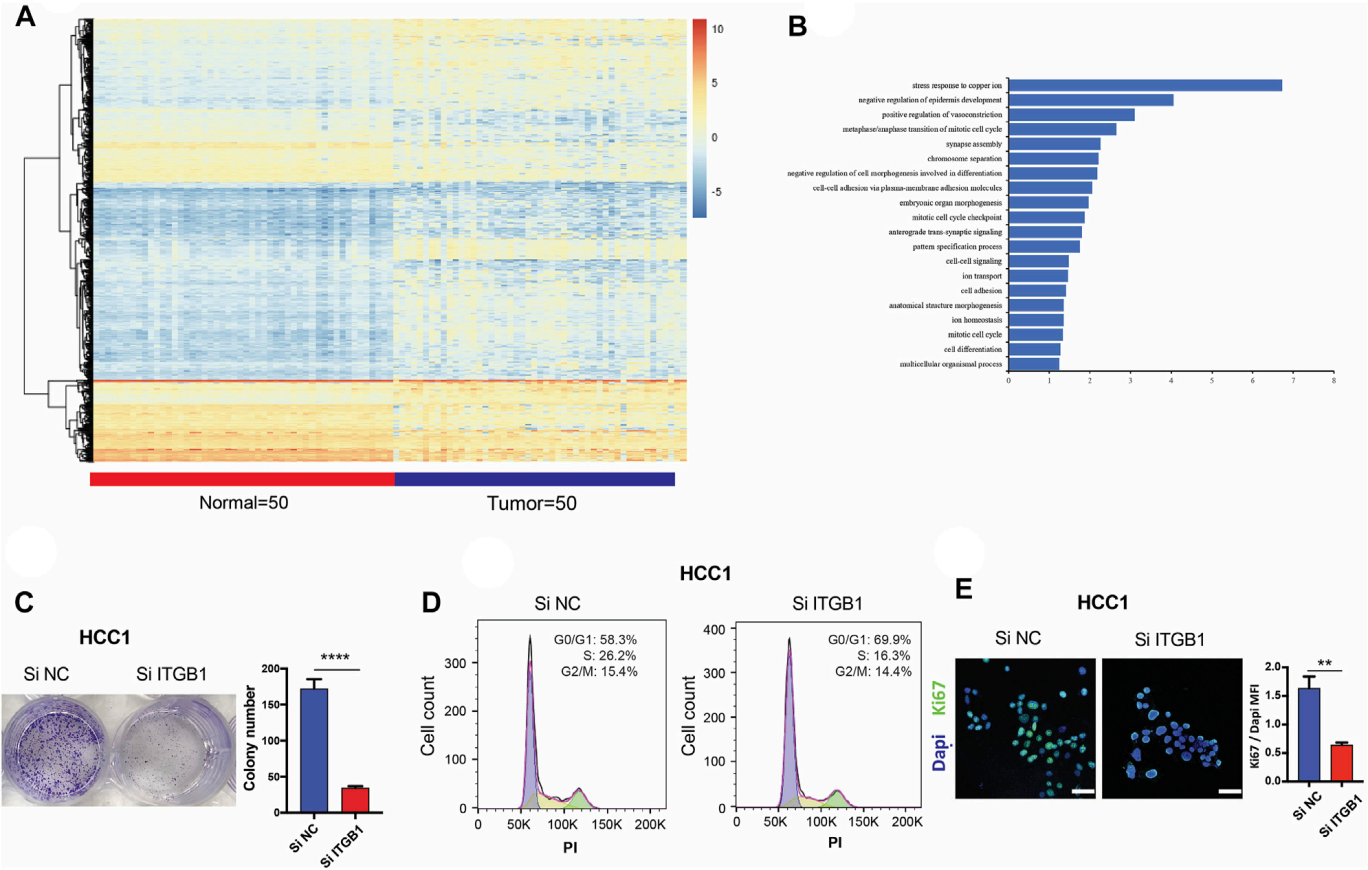
ITGB1 promoted the progression of HCC by facilitating mitotic cell cycle. **(A)** Heatmap of differentially expressed genes in LIHC patients (*n* = 50) and normal samples (*n* = 50) derived from TCGA data. **(B)** TBtools software was used for functional enrichment analysis of differentially expressed genes derived from TCGA data, whose significance threshold of *p*-value was less than 0.05. **(C)** Representative images **(left)** and quantification **(right)** of colony formation assays for the measurement of proliferation of HCC1 cells treated with ITGB1 siRNA after 6 days. **(D)** Cell cycle distribution of HCC1 cells treated with ITGB1 siRNA was determined by flow cytometry with propidium iodide staining. **(E)** Representative images **(left)** and quantification **(right)** of Ki67 expressed in HCC1 cells treated with ITGB1 siRNA after 3 days. Samples were probed by anti-Ki67 (green) Ab and counterstained by DAPI (blue). Scale bar = 50 µm. Values are presented in mean ± SD. **p < 0.01, ****p < 0.0001.

In an attempt to elucidate molecular pathway driven by ITGB1 for enhancing HCC progression, we analyzed the mRNA expression profile of HCC patients (*N* = 50) in comparison with normal samples (*N* = 50) by utilizing TCGA dataset. We identified 4,111 differentially expressed genes in total, among which 3,027 genes were upregulated, while 1,084 genes were downregulated (Figure 4A and Supplementary Table S3). Next, the functional role of ITGB1 in HCC was analyzed by using differentially expressed genes (Figure 4B). Noteworthy, ITGB1 directly participated in modulating mitotic cell cycle pathway, whose aberration has been defined as a hallmark of cancer. Therefore, in order to clarify the role of ITGB1, ITGB1 expression in HCC cells was inhibited by siRNA. The results of colony formation assay verified that ITGB1 deficiency dramatically reduced HCC cell colony formation (Figure 4C and Supplementary Figure S3A), revealing that ITGB1 promoted HCC cell proliferation. Moreover, using flow cytometry, we examined cell cycle distribution in ITGB1-inhibited HCC cells and found these cells were arrested in G1 phase in comparison with control cells (Figure 4D and Supplementary Figure S3B). Next, we performed confocal analysis of Ki67 (marker of proliferating cells) and obtained similar results that the deficiency of ITGB1 halted the proliferation of HCC cells (Figure 4E and Supplementary Figure S3C). Consistently, siRNA for ITGB1 greatly suppressed HCC cell viability (Supplementary Figure S3D). Additionally, we estimated the expression of Ki67 (marker of proliferating cells) and regulators of cell cycle progression, including cell division cycle 42 (CDC42), cyclin A2 (CCNA2), cyclin B1 (CCNB1), cyclin D1 (CCND1), and cyclin E1 (CCNE1) in TCGA database, and found that all the genes except CCND1 were significantly upregulated in HCC tissues compared with normal controls (Supplementary Figure S4A). Furthermore, we observed that ITGB1 expression was positively correlated with the mRNA expression of Ki67, CDC42, cyclin A2, cyclinB1, cyclin D1, and cyclinE1, respectively, by analyzing TCGA data (Supplementary Figure S4B). Altogether, these findings highlighted that ITGB1 played a vital role in fostering HCC growth *via* activating cell cycle pathway.

### Identification of the Potential Molecules That Are Involved in Integrin β1-Driven Cell Cycle Process

**FIGURE 5:**
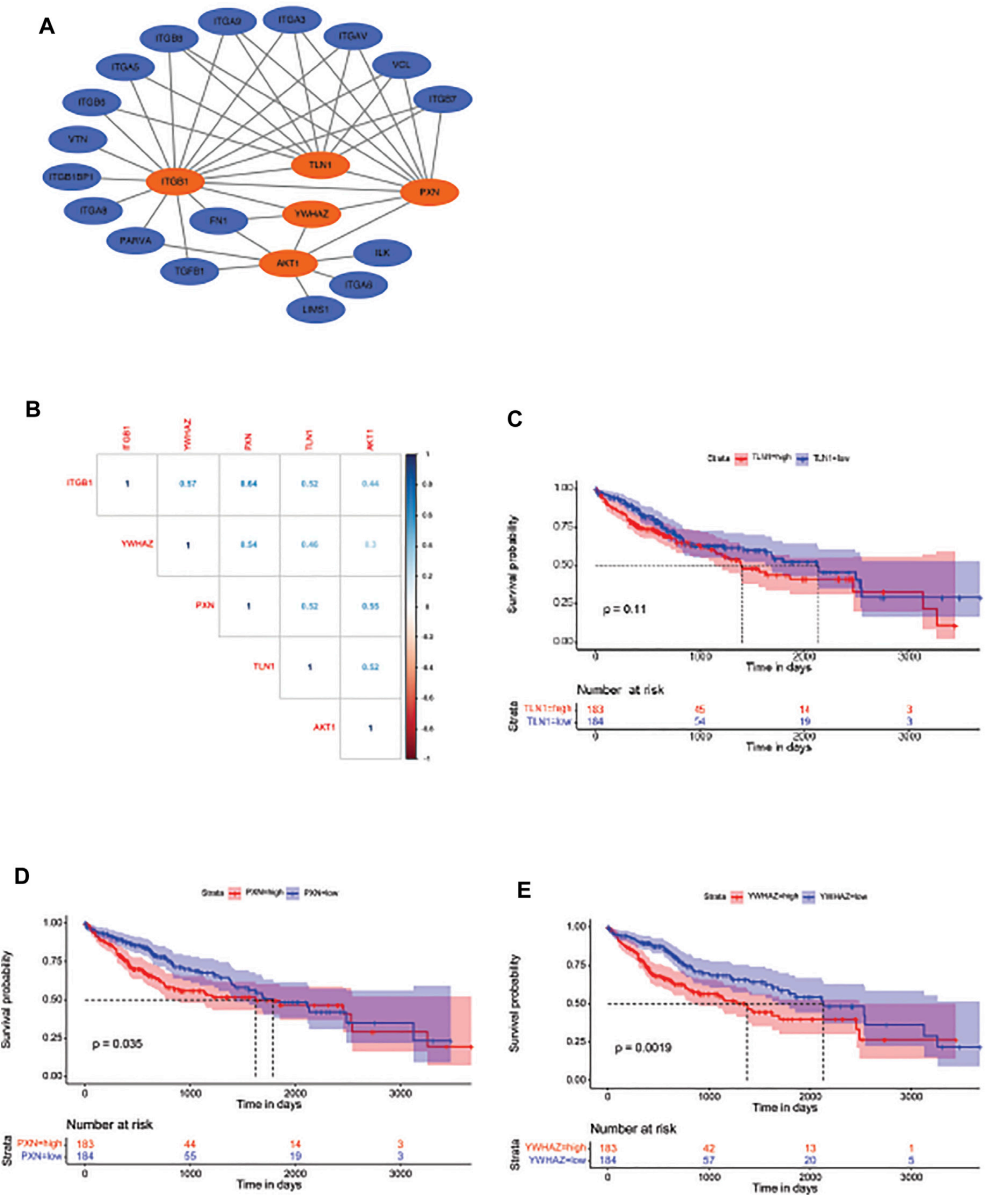
Identification of the potential molecules that are involved in ITGB1-driven cell cycle process. **(A)** Gene set enrichment analysis for the identified genes involved in mitotic cell cycle signal pathway. **(B)** The expression correlation between ITGB1 and YWHAZ, PXN, TLN1, and AKT. **(C)** Kaplan–Meier overall survival curve was shown according to high and low expression of TLN1 in LIHC patients derived from TCGA data. **(D)** Kaplan–Meier overall survival curve is shown according to high and low expression of PXN in LIHC patients derived from TCGA data. **(E)** Kaplan–Meier overall survival curve is shown according to high and low expression of YWHAZ in LIHC patients derived from TCGA data.

To further elucidate ITGB1-regulated cell cycle pathway, we analyzed the PPI network of ITGB1 in STRING database and screened the potential proteins that strongly interacted with ITGB1 (Figure 5A). Intriguingly, we observed that talin-1 (TLN1), PXN, YWHAZ, and AKT, which were all differentially expressed genes involved in the ITGB1-regulated cell cycle pathway, might also interact with ITGB1 (Figure 5A). In addition, the correlogram, which exhibited the relationships between variables, illustrated that ITGB1 was positively associated with TLN1, PXN, YWHAZ, and AKT (Figure 5B). Furthermore, we also evaluated the influence of the expression of TLN1, PXN, and YWHAZ on the overall survival in HCC patients. No significant difference was detected in the overall survival between TLN1-high and -low groups (Figure 5C). By contrast, the overall survival of HCC patients with high levels of PXN and YWHAZ was significantly decreased as compared to patients with a low expression of these two proteins, respectively (Figure 5D, E). These results indicated that PXN and YWHAZ might serve as critical molecules involved in ITGB1-regulated cell cycle process.

### Integrin β1 Facilitated Hepatocellular Carcinoma Cell Cycle Progression *via* Regulating PXN/YWHAZ/AKT Pathway

**FIGURE 6:**
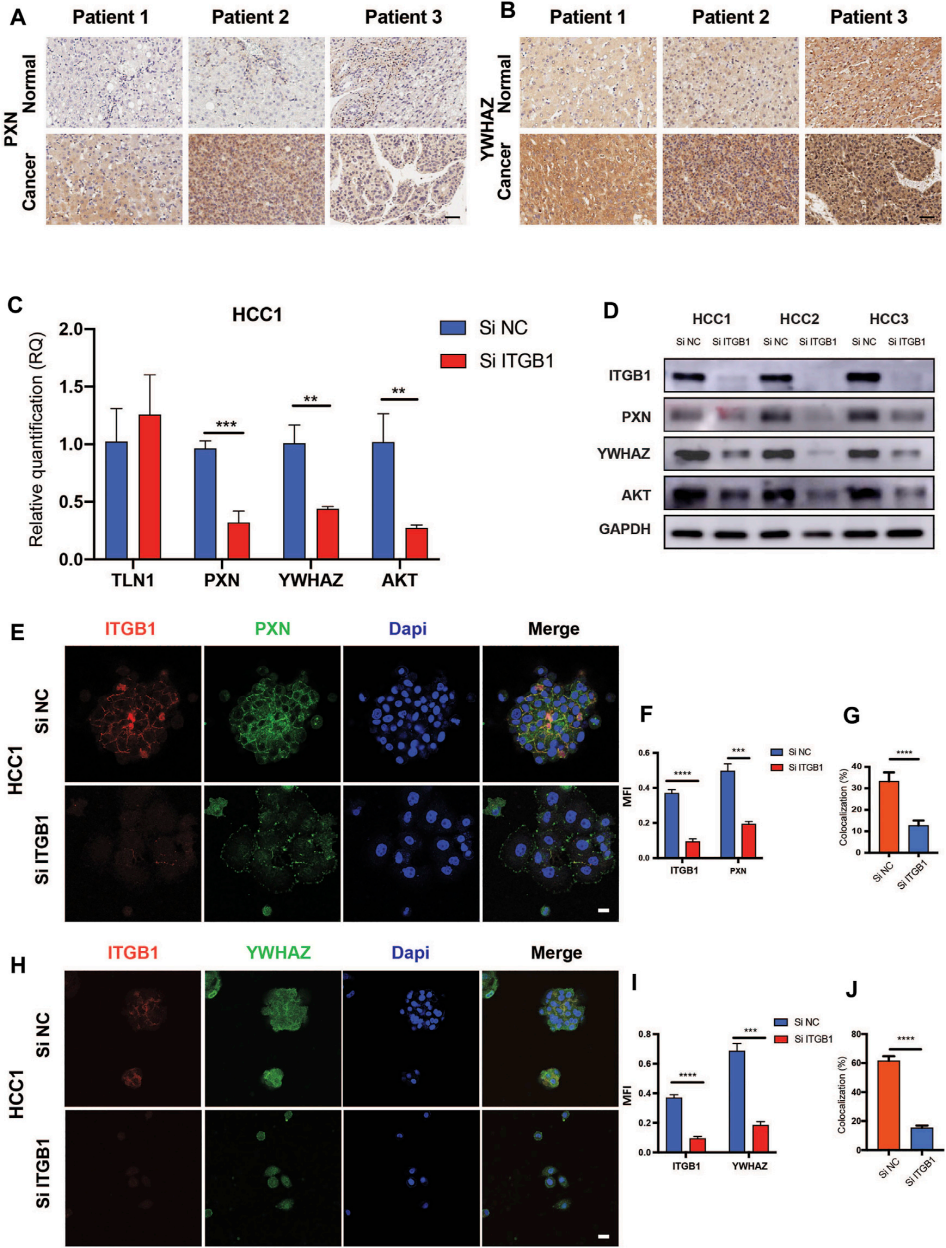
ITGB1 facilitated HCC cell cycle progression *via* regulating PXN/YWHAZ/AKT pathway. **(A)** Representative images of PXN immunohistochemical staining in paired tumor-adjacent normal tissues (*n* = 10) and LIHC tissues (*n* = 10). Scale bar =50 µm. **(B)** Representative images of YWHAZ immunohistochemical staining in paired tumor-adjacent normal tissues (*n* = 10) and LIHC tissues (*n* = 10). Scale bar = 50 µm. **(C)** qRT-PCR a alysis for the expression of TLN1, PXN, YWHAZ, and AKT in HCC1 cells treated with ITGB1 siRNA was assessed. **(D)** Western blot was used to evaluate the protein expression of PXN, YWHAZ, and AKT in HCC1, 2, and 3 cells treated with ITGB1 siRNA. **(E)** Representative confocal images of PXN and ITGB1 in HCC1 cells treated with ITGB1 siRNA. Scale bar = 100 µm. **(F)** The quantification of protein expression of ITGB1 and PXN, respectively, and **(G)** co-staining of PXN and ITGB1 by immunostaining analysis. Samples were probed by anti-PXN (green) Ab and anti-ITGB1 (red) Ab and counterstained by DAPI (blue). **(H)** Representative confocal images of YWHAZ and ITGB1 in HCC1 cells treated with ITGB1 siRNA. Scale bar = 100 µm. **(I)** The quantification of protein expression of ITGB1 and YWHAZ, respectively, and **(J)** co-staining of YWHAZ and ITGB1 by immunostaining analysis. Samples were probed by anti-YWHAZ (green) Ab and anti-ITGB1 (red) Ab and counterstained by DAPI (blue). Values are presented in mean ± SD. **p < 0.01, ***p < 0.001, ****p < 0.0001.

**FIGURE 7:**
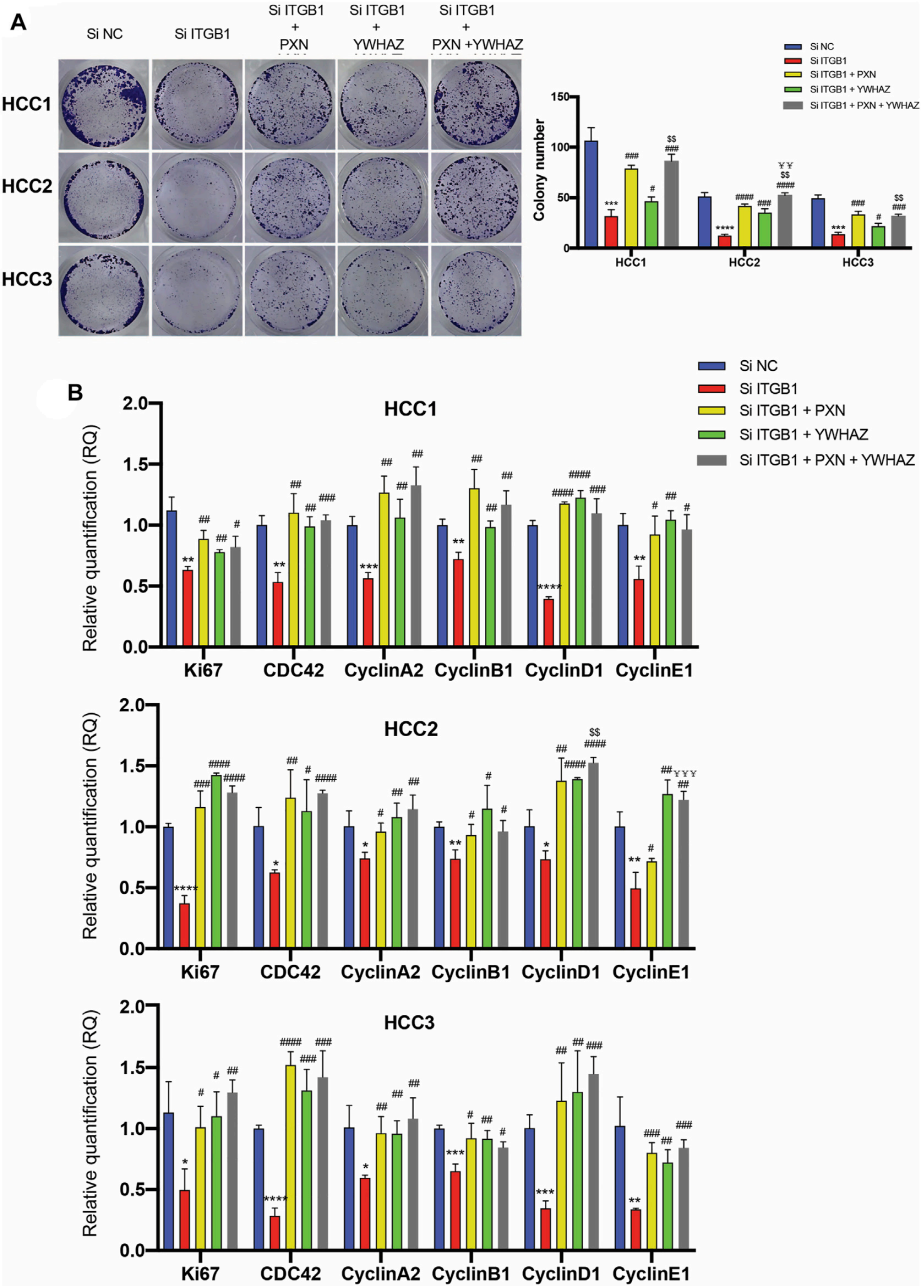
ITGB1 facilitated HCC cell cycle progression *via* regulating PXN/YWHAZ/AKT pathway. **(A)** Representative images **(left)** and quantification **(right)** of colony formation assays for the measurement of proliferation of HCC cells treated with siITGB1, siITGB1 plus PXN, siITGB1 plus YWHAZ, and siITGB1 plus PXN plus YWHAZ after 6 days. **(B)** qRT-PCR analysis for expression of Ki67, CDC42, cyclin A2, cyclin B1, cyclin D1, and cyclin E1 in HCC cells treated with siITGB1, siITGB1 plus PXN, siITGB1 plus YWHAZ, and siITGB1 plus PXN plus YWHAZ. Values are presented in mean±SD. *Used for the comparison of statistical significance between siNC and siITGB1 groups. ^#^Used for the comparison of statistical significance of siITGB1 compared with siITGB1 plus PXN, siITGB1 plus YWHAZ, and siITGB1 plus PXN plus YWHAZ groups. ^$^Used for the comparison of statistical significance between siITGB1 plus YWHAZ and siITGB1 plus PXN plus YWHAZ groups. ^¥^Used for the comparison of statistical significance between siITGB1 plus PXN and siITGB1 plus PXN plus YWHAZ groups. *p < 0.05, **p < 0.01, ***p < 0.001, ****p < 0.0001. ^#^ *p* < 0.05, ^##^ *p* < 0.01, ^###^ *p* < 0.001, ^####^ *p* < 0.0001, ^$$^ *p* < 0.01. ^¥¥^ *p* < 0.01, ^¥¥¥^ *p* < 0.001.

According to the analysis that high levels of PXN and YWHAZ were significantly associated with poor prognosis in HCC patients, we decided to initially examine their protein expression. As expected, IHC analysis revealed that both PXN and YWHAZ were notably upregulated in HCC tumor specimens in contrast with normal peritumoral tissues (Figure 6A, B). In an attempt to clarify whether the expression of PXN and YWHAZ is affected by ITGB1, we silenced ITGB1 expression in primary HCC cells by transfecting siRNA against ITGB1. We found that the mRNA levels of TLN1 was unchanged in the siITGB1 group, while the knockdown of ITGB1 induced a strongly repressed expression of PXN, YWHAZ, and AKT as shown by qRT-PCR analysis (Figure 6C and Supplementary Figure S5A). Similar results were obtained by measuring their protein expression levels (Figure 6D). Moreover, immunostaining results further confirmed that PXN and YWHAZ were both declined in siITGB1-treated HCC cells (Figure 6E, F, H, I; Supplementary Figure S5B–E; Supplementary Figure S6A–D). Interestingly, high percentage of colocalization between ITGB1 and PXN or YWHAZ was observed in HCC cells, indicating that ITGB1 might interact with PXN and YWHAZ (Figure 6E, G, H, J, Supplementary Figure S5A, B, E, F; Supplementary Figure S6A, B, E, F). Moreover, we designed rescue experiments to verify that PXN and YWHAZ serve as vital downstream target genes of ITGB1. First, by performing colony formation assays, we observed a profoundly reduced colony number in the siITGB1-treated group versus the siNC group. Of note, the addition of PXN or YWHAZ protein alone was already capable to markedly reverse the decreased colony number of HCC cells treated with siITGB1 (Figure 7A). Furthermore, we conducted qRT-PCR analysis for examining the effects of PXN and YWHAZ on the expression of mitotic cell cycle markers (Ki67, CDC42, cyclin A2, cyclin B1, cyclin D1, and cyclin E1). As expected, siITGB1 profoundly down-regulated the mRNA expression of these genes. In addition, PXN or YWHAZ treatment alone efficiently reverted the inhibited expression of all mitotic cell cycle markers induce by siITGB1 (Figure 7B). Together, these data illustrated that ITGB1 enhanced HCC cell cycle progression through the activation of PXN/YWHAZ/AKT pathway.

### Integrin β1 Expression Modulated Hepatocellular Carcinoma Tumor Growth *In Vivo*

**FIGURE 8:**
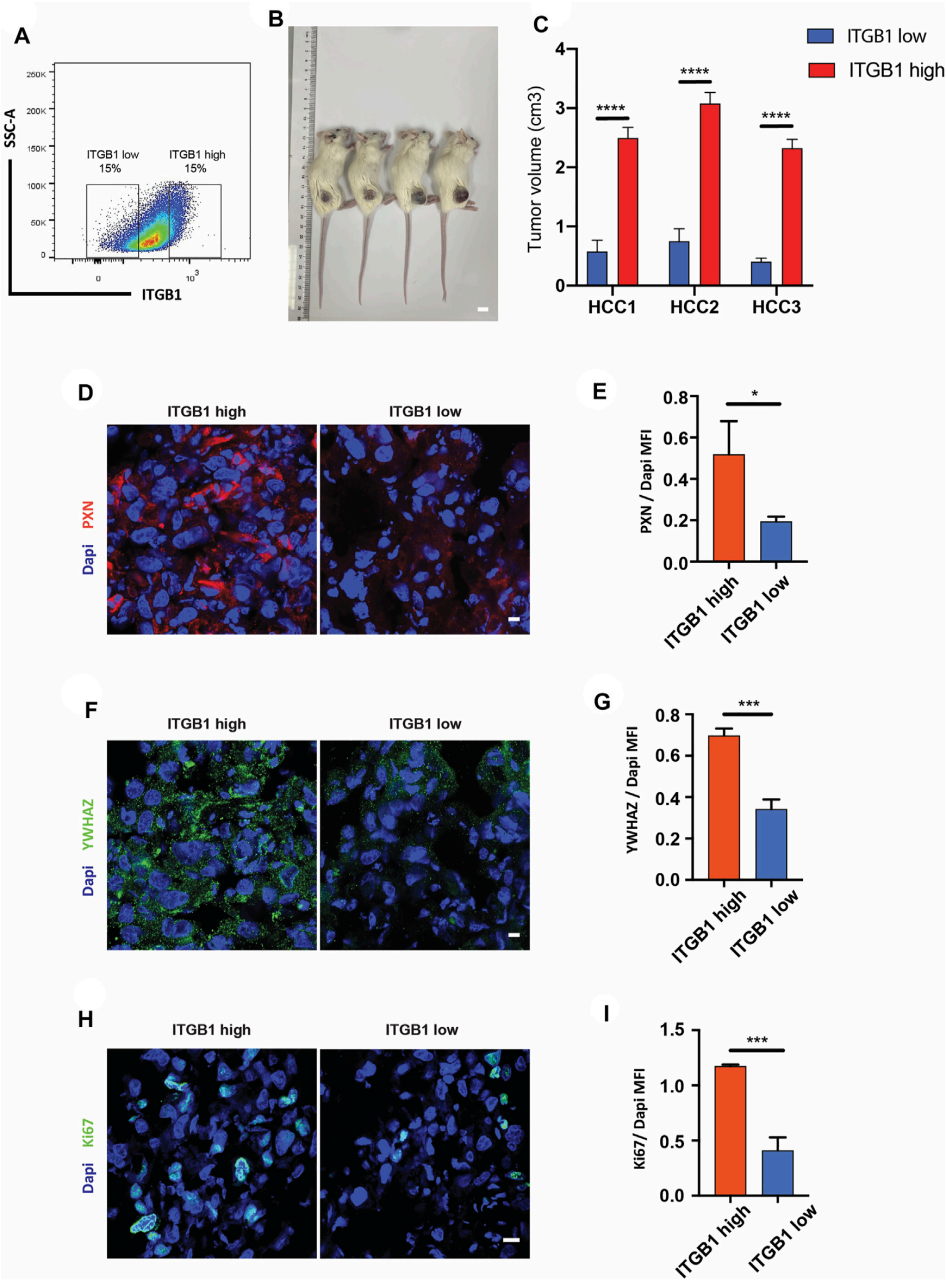
ITGB1 expression modulated HCC tumor growth *in vivo.* **(A)** Gating strategy for isolating ITGB1^high^ and ITGB1^low^cells from HCC cells. **(B)** Representative images of subcutaneous tumors formed by ITGB1^low^ or ITGB1^high^ HCC1 cells in mice. Scale bar = 1 cm. **(C)** The quantification of tumor volume of subcutaneous tumors of HCC cells (*n* = 5 per group). **(D)** Representative confocal images of PXN in tissues from ITGB1^high^ and ITGB1^low^ HCC1 cell-formed tumors. Scale bar = 100 µm. **(E)** The quantification of the protein expression of PXN by immunostaining analysis. Samples were probed by anti-PXN (red) Ab and counterstained by DAPI (blue). **(F)** Representative confocal images of YWHAZ in tissues from ITGB1^high^ and ITGB1^low^ HCC1 cell-formed tumors. Scale bar = 100 µm. **(G)** The quantification of protein expression of YWHAZ by immunostaining analysis. Samples were probed by anti-YWHAZ (green) Ab and counterstained by DAPI (blue). **(H)** Representative confocal images of Ki67 in tissues from ITGB1^high^ and ITGB1^low^ HCC1 cell-formed tumors. Scale bar = 100 µm. **(I)** The quantification of the protein expression of Ki67 by immunostaining analysis. Samples were probed by anti-Ki67 (green) Ab and counterstained by DAPI (blue). Values are presented in mean ± SD. *p < 0.05, ***p < 0.001, ****p < 0.0001.

Stemming from *in vitro* findings, we became interested in evaluating the influence of ITGB1 on HCC tumor growth. To this end, we first checked the ITGB1 expression profile in primary HCC cells using flow cytometry. As shown in Figure 8A, primary HCC cells expressed a wide range of protein level of ITGB1. Therefore, ITGB1^high^- and ITGB1^low^-expressing populations were isolated from those HCC cells and subcutaneously inoculated into NSG mice (Figure 8A). After 1 month, we noticed that ITGB1^high^-expressing cells formed more rapidly growing tumors than ITGB1^low^-expressing cells (Figure 8B, C). In line with the high expression of ITGB1, the protein levels of PXN and YWHAZ were both much higher in ITGB1^high^ tumors as examined by immunostaining (Figure 8D–G). Furthermore, the immunostaining analysis of Ki67, which is a marker of proliferating cells, demonstrated more proliferating cells in ITGB1^high^ tumors than ITGB1^low^ tumors (Figure 8H, I). Thus, our experiments provided evidence that ITGB1 plays an essential role in accelerating HCC cell proliferation, ultimately hastening HCC tumor growth.

## Discussion

ITGB1, as the most abundantly expressed beta integrin, has been reported to be overexpressed in HCC tissues (Jiang et al., 2015). However, little is known concerning the role of ITGB1 in modulating the onset and progression of HCC. In the present study, for the first time, we examined the influence and the mechanisms of action of ITGB1 in both patient-derived primary HCC cells and primary tumor cell-derived xenograft model. Herein, we demonstrated that ITGB1 enhanced HCC tumor progression *via* PXN/YWHAZ/AKT signaling pathways, which confers an increased growth ability to HCC tumors.

It has been previously documented that the expression level of ITGB1 in HCC tumor tissues was significantly higher than that in peritumoral tissues (Liu et al., 2002; Zhao et al., 2010; Jiang et al., 2015). Consistently, we also observed an upregulated expression of ITGB1 in HCC tumor tissues as compared with matched non-tumor tissues. Additionally, on the basis of TCGA data, we found that a high level of ITGB1 expression in HCC patients was strongly associated with high pathological stages as well as poor survival, indicating that ITGB1 might potentially serve as a poor prognostic biomarker in HCC progression.

Gene copy number alterations and DNA methylation have been identified as specific mechanisms that modulate the expression of tumorigenesis-associated genes (Fan et al., 2018; Bhattacharya et al., 2020). To date, the mechanism that is responsible for abnormal ITGB1 expression in HCC still remains an enigma. In the present study, we did not detect a significant difference in ITGB1 gene copy number between normal and HCC samples, based on the analysis of TCGA database. On the contrary, we showed that ITGB1 overexpression was notably correlated with declined DNA methylation, which was further confirmed in paired HCC and peritumoral tissues. Indeed, the role of DNA methylation displayed in regulating ITGB1 expression has been previously described in coronary artery disease (Miao et al., 2019). Together, these findings revealed that DNA methylation may contribute to ITGB1 dysfunction in HCC progression.

Recently, ITGB1 has been characterized to induce EMT in HCC cell lines through ILK-AKT-mTOR signaling cascade, leading to more aggressive and drug-resistant phenotypes in HCC cells (Jiang et al., 2015). Consistent with these data, we observed that siRNA-modulated silencing of ITGB1 was capable to impair HCC cell migration, viability, and drug resistance in primary HCC cells. Furthermore, siRNA targeting ITGB1 efficiently alleviated the phosphorylation and protein maturation of MET, which triggers tumor formation and growth (Johnson et al., 1995; Bogorad et al., 2014). However, in this study, the authors utilized a MET/ΔN90-β-catenin-driven mouse model of HCC, in which multiple signaling pathways might be changed as compared with spontaneous HCC. Of interest, our combinatory analysis of Gene Ontology (GO) enrichment and PPI network indicated that ITGB1 directly accelerated HCC cell cycle progression by activating PXN/YWHAZ/AKT signaling pathway, based on TCGA data.

Uncontrolled cell proliferation, which is controlled by cell cycle, is one of the main reasons for high mortality of HCC (Collins et al., 1997; Xia et al., 2019). In fact, our experimental data corroborated that ITGB1 was essential for cell proliferation. Accumulating evidence illustrated that ITGB1 sustains cell proliferation through the upregulation of cyclin D1 in colorectal cancer cells and satellite cells (Song et al., 2014; Rozo et al., 2016). In agreement, we observed that the knockdown of ITGB1 markedly retarded cell cycle progression and was accompanied by a strong reduction in cyclin D1, cyclin A2, cyclin B1, cyclin E1, and CDC42 expression.

PXN, which is an intracellular adaptor protein connecting integrins for signal transduction into the cell, has been estimated to be upregulated in various cancer types, including lung cancer, gastric cancer, and breast cancer (Du et al., 2016; Lisiak et al., 2017). Moreover, recent studies revealed that PXN displayed a pro-tumorigenic function during HCC progression (Yuan et al., 2017). Of note, Lisiak et al. demonstrated that the derivatives of oleanolic acid could impair the migration and invasion of human breast cancer cells by simultaneously suppressing the expression of ITGB1 and PXN, indicating a possible correlation between ITGB1 and PXN expression (Lisiak et al., 2017). In line with the above finding, our observation verified that ITGB1 deficiency induced the downregulated expression of PXN in both HCC cells and HCC tumors.

Noteworthy, YWHAZ, which is a central hub protein regulating multiple biological processes, was also predicted to be implicated in ITGB1-mediated HCC tumor growth, based on our comprehensive analysis. A growing body of literature has proved that YWHAZ is overexpressed in a wide range of tumor types, such as lung cancer, breast cancer, colorectal cancer, and HCC (Gan et al., 2020). In addition, YWHAZ has been well defined to play a crucial role in modulating cell cycle processes by controlling cellular senescence and G2/M checkpoint (Gardino and Yaffe, 2011). Herein, our study for the first time showed that ITGB1 might interact with YWHAZ, and ITGB1 suppression resulted in repressed expression of YWHAZ. Additionally, the data of rescue experiments further confirmed that PXN and YWHAZ were both involved in ITGB1-regulated cell cycle process, suggesting that ITGB1 promotes HCC progression *via* PXN and YWHAZ that might operate concurrently.

Owing to acting as an ECM receptor, ITGB1 has been identified to be a promising target to develop novel therapeutic strategies for HCC. In the present study, by means of TCGA database analysis and experimental data acquired in primary HCC cells, we illustrated that ITGB1 modulated HCC cell proliferation *via* PXN/YWHAZ/AKT axis, which could be a potential target for HCC treatment. Although our research, to a certain extent, provides more clinically relevant significance, there are still several questions remaining concerning the function of ITGB1/PXN/YWHAZ/AKT signaling axis. For instance, does this axis also exert similar impacts on other tumor types? Are other mechanisms involved in ITGB1-mediated HCC cell proliferation? Therefore, further investigation is required to answer above questions, which will be beneficial to illuminate the tumorigenesis of HCC.

## Conclusion

In summary, this study highlights the novel mechanisms underlying the ITGB1-mediated HCC progression by analyzing TCGA database and verifying in HCC primary cells. We uncovered, for the first time, that PXN and YWHAZ are direct downstream targets of ITGB1. During HCC development, PXN and YWHAZ may be both activated by the upregulation of ITGB1 and cooperate to accelerate the cell cycle process. Our data indicates that ITGB1/PXN/YWHAZ axis is a promising target to develop effective therapeutic strategies for HCC.

## Data Availability

Publicly available datasets were analyzed in this study. This data can be found here: https://www.cancer.gov/about-nci/organization/ccg/research/structural-genomics/tcga.

## Supplementary Material

Supplementary Figure 1 Representative image of methylation-specific PCR products for ITGB1 gene in paired tumor-adjacent normal tissues (*n* = 7) and LIHC tissues (*n* = 7). M, methylated sequence; U, unmethylated sequence; T, tumor; N, tumor-adjacent normal tissues.

Supplementary Figure 2
**(A)** The representative confocal images of AFP in HCC1, 2, and 3 cells derived from LIHC tissues. Samples were probed by anti-AFP (green) Ab and counterstained by DAPI (blue). **(B)** The representative confocal images of ASGPR in HCC1, 2, and 3 cells derived from LIHC tissues. Samples were probed by anti-ASGPR (green) Ab and counterstained by DAPI (blue). Scale bar = 50 µm.

Supplementary Figure 3 ITGB1 promoted the progression of HCC by facilitating mitotic cell cycle. **(A)** Representative images (upper) and quantification (down) of colony formation assays for the measurement of proliferation of HCC2 and 3 cells treated with ITGB1 siRNA after 6 days. **(B)** Cell cycle distribution of HCC2 and 3 cells treated with ITGB1 siRNA was determined by flow cytometry with propidium iodide staining. **(C)** Representative images **(left)** and quantification **(right)** of Ki67 expressed in HCC2 and 3 cells treated with ITGB1 siRNA after 3 days. Samples were probed by anti-Ki67 (green) Ab and counterstained by DAPI (blue). Scale bar = 50 µm. **(D)** The cell viability of HCC1, 2, and 3 cells treated with ITGB1 siRNA was determined by PrestoBlue at 24, 48, and 72 h. Values are represented in mean ± SD. **p* < 0.05, ***p* < 0.01, ****p* < 0.001, *****p* < 0.0001.

Supplementary Figure 4 ITGB1 promoted the progression of HCC by facilitating mitotic cell cycle. **(A)** mRNA expression of MKI67, CDC42, CCNA2, CCNB1, CCND1, and CCNE1 in normal tissues (*n* = 50) and LIHC tissues (*n* = 369) derived from TCGA data. **(B)** The correlation analysis between ITGB1 expression and MKI67, CDC42, CCNA2, CCNB1, CCND1, and CCNE1 expression derived from TCGA data.

Supplementary Figure 5 ITGB1 facilitated HCC cell cycle progression *via* regulating PXN/YWHAZ/AKT pathway. **(A)** qRT-PCR analysis for the expression of TLN1, PXN, YWHAZ, and AKT in HCC2 and 3 cells treated with ITGB1 siRNA. **(B and C)** Representative confocal images of PXN and ITGB1 in HCC2 and 3 cells treated with ITGB1 siRNA. Scale bar =100 µm. **(D and E)** The quantification of protein expression of PXN and ITGB1, respectively, and **(F and G)** co-staining of PXN and ITGB1 by immunostaining analysis. Samples were probed by anti-PXN (green) Ab and anti-ITGB1 (red) Ab and counterstained by DAPI (blue). Values are presented in mean ± SD. ****p* < 0.001, *****p* < 0.0001.

Supplementary Figure 6 ITGB1 facilitated HCC cell cycle progression *via* regulating PXN/YWHAZ/AKT pathway. **(A and B)** Representative confocal images of YWHAZ and ITGB1 in HCC2 and 3 cells treated with ITGB1 siRNA. Scale bar = 100 µm. **(C and D)** The quantification of protein expression of YWHAZ and ITGB1, respectively, and **(E and F)** co-staining of YWHAZ and ITGB1 by immunostaining analysis. Samples were probed by anti-YWHAZ (green) Ab and anti-ITGB1 (red) Ab and counterstained by DAPI (blue). Values are presented in mean ± SD. ****p* < 0.001, *****p* < 0.0001.

## Abbreviations

AKT, protein kinase B; ASGPR, asialoglycoprotein receptor cyclin; CCNA2, cyclin A2; CCNB1, cyclin B1; CCND1, cyclin D1; CCNE1, cyclin E1; CDC42, cell division cycle 42; ECM, extracellular matrix; EMT, epithelial–mesenchymal transition; FAK, focal adhesion kinase; GO, Gene Ontology; HCC, hepatocellular carcinoma; LIHC, liver hepatocellular carcinoma; IHC, immunohistochemistry; ITGB1, integrin β1; MSP, methylation-specific PCR; NSG, NOD scid gamma; PXN, paxillin; PPI, protein–protein interaction; qRT-PCR, quantitative real-time polymerase chain reaction; RQ, relative quantity; SiRNA, small interfering RNA; TLN1, talin-1; TCGA, The Cancer Genome Atlas; YWHAZ, 14-3-3 protein zeta.
