# Supplementary figures and images for: ITGB1 Drives Hepatocellular Carcinoma Progression by Modulating Cell Cycle Process Through PXN/YWHAZ/AKT Pathways
Source: Front Cell Dev Biol. 2021 Dec 17;9:711149. doi: 10.3389/fcell.2021.711149 (PMC8718767; doi:10.3389/fcell.2021.711149)

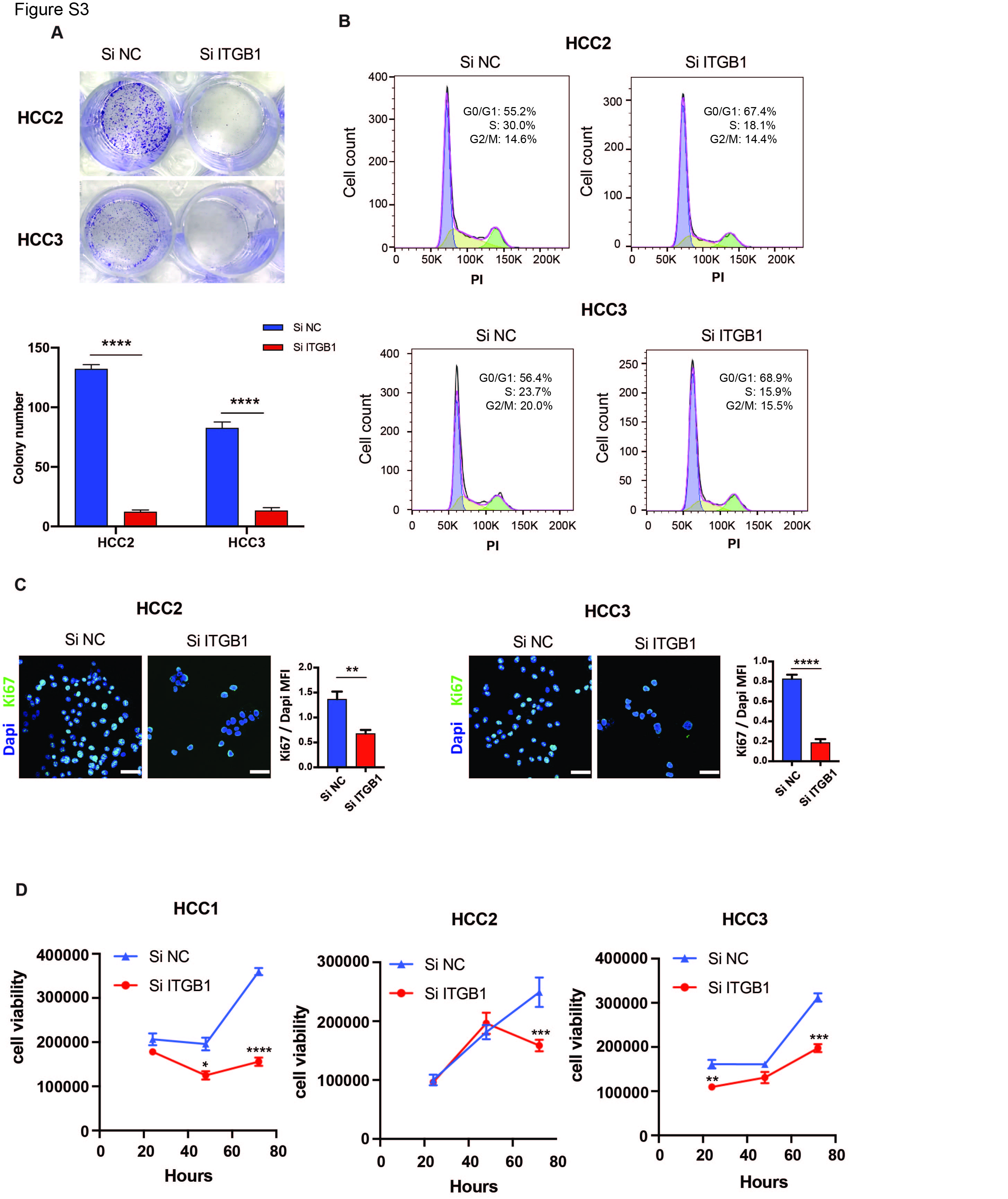

Supplement: Supplementary file 2 [file Image3.jpeg]

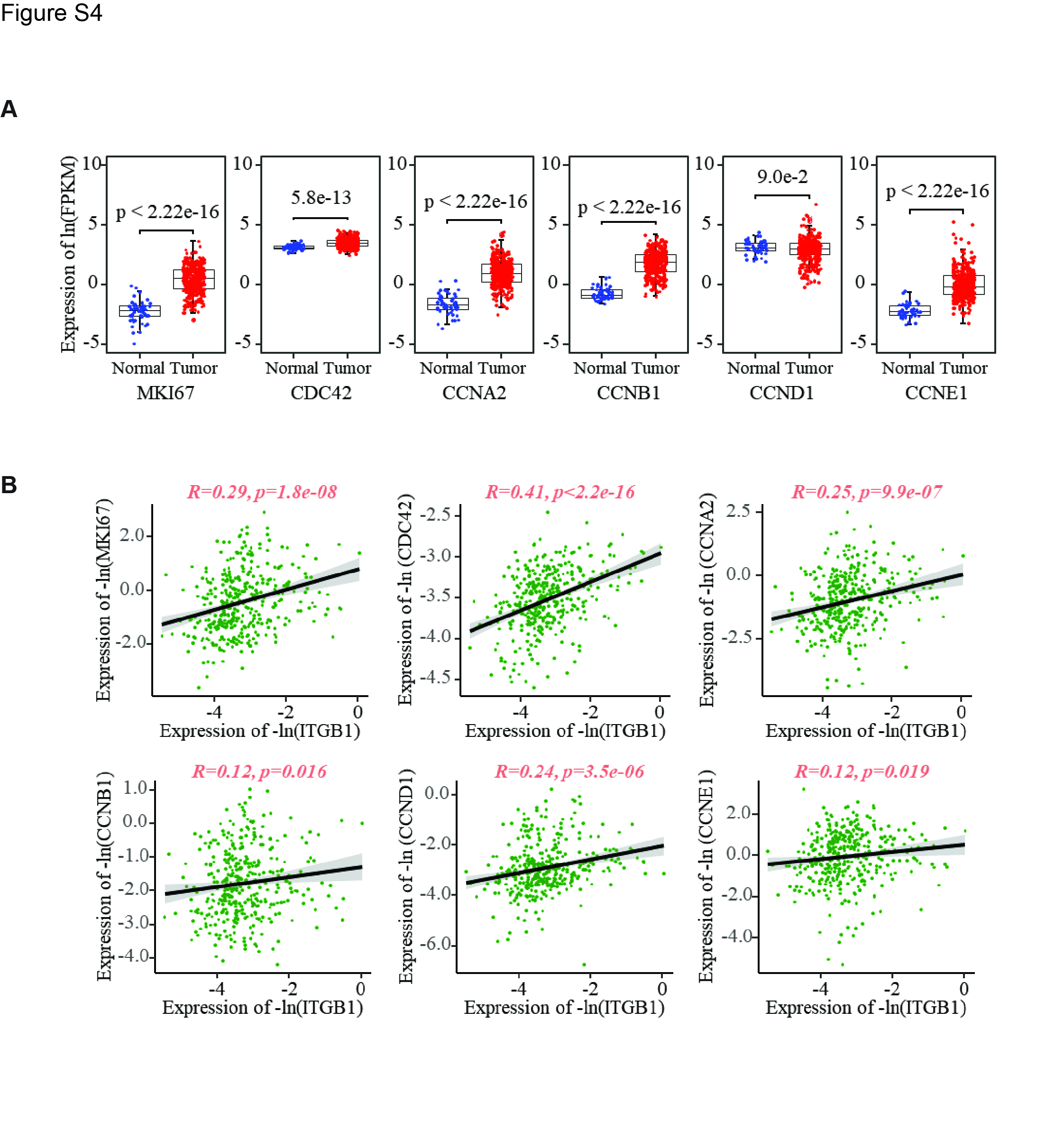

Supplement: Supplementary file 5 [file Image4.tif]

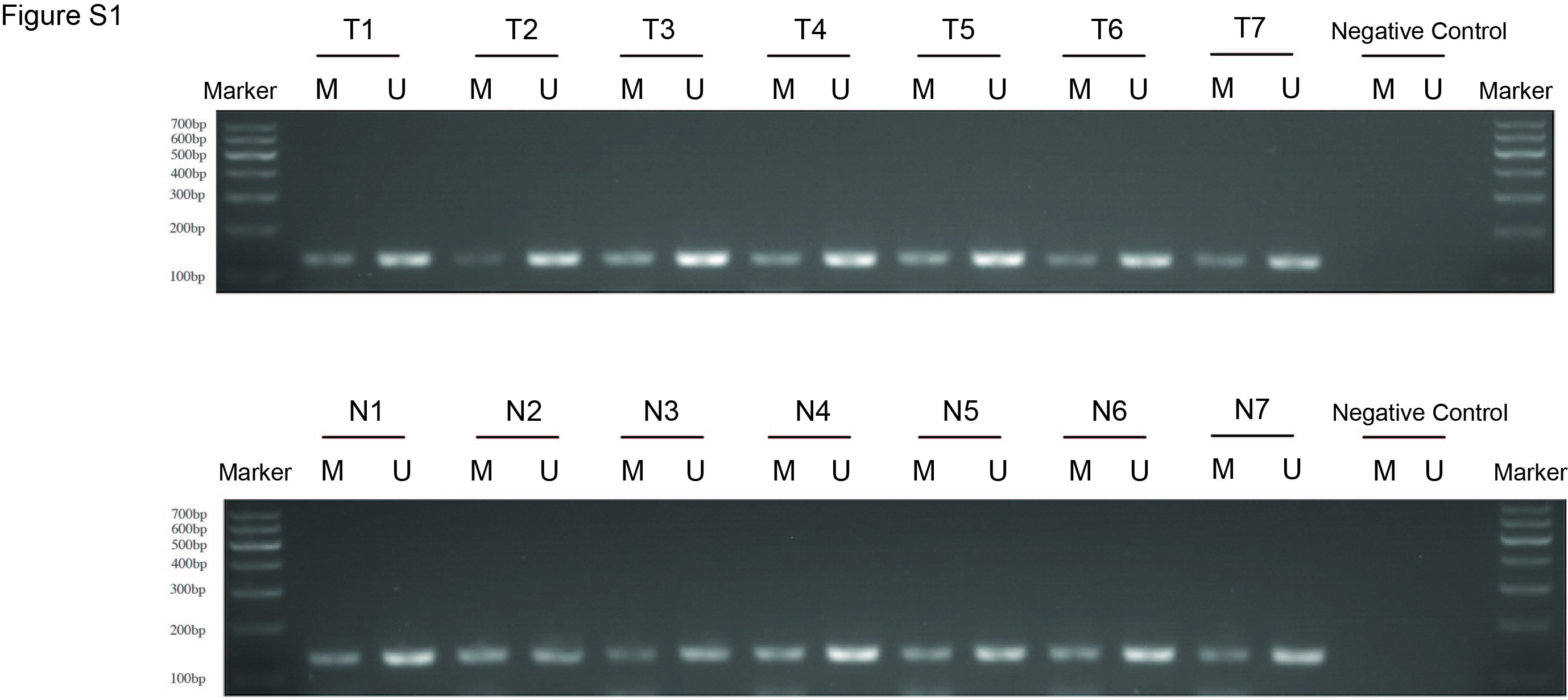

Supplement: Supplementary file 6 [file Image1.jpeg]

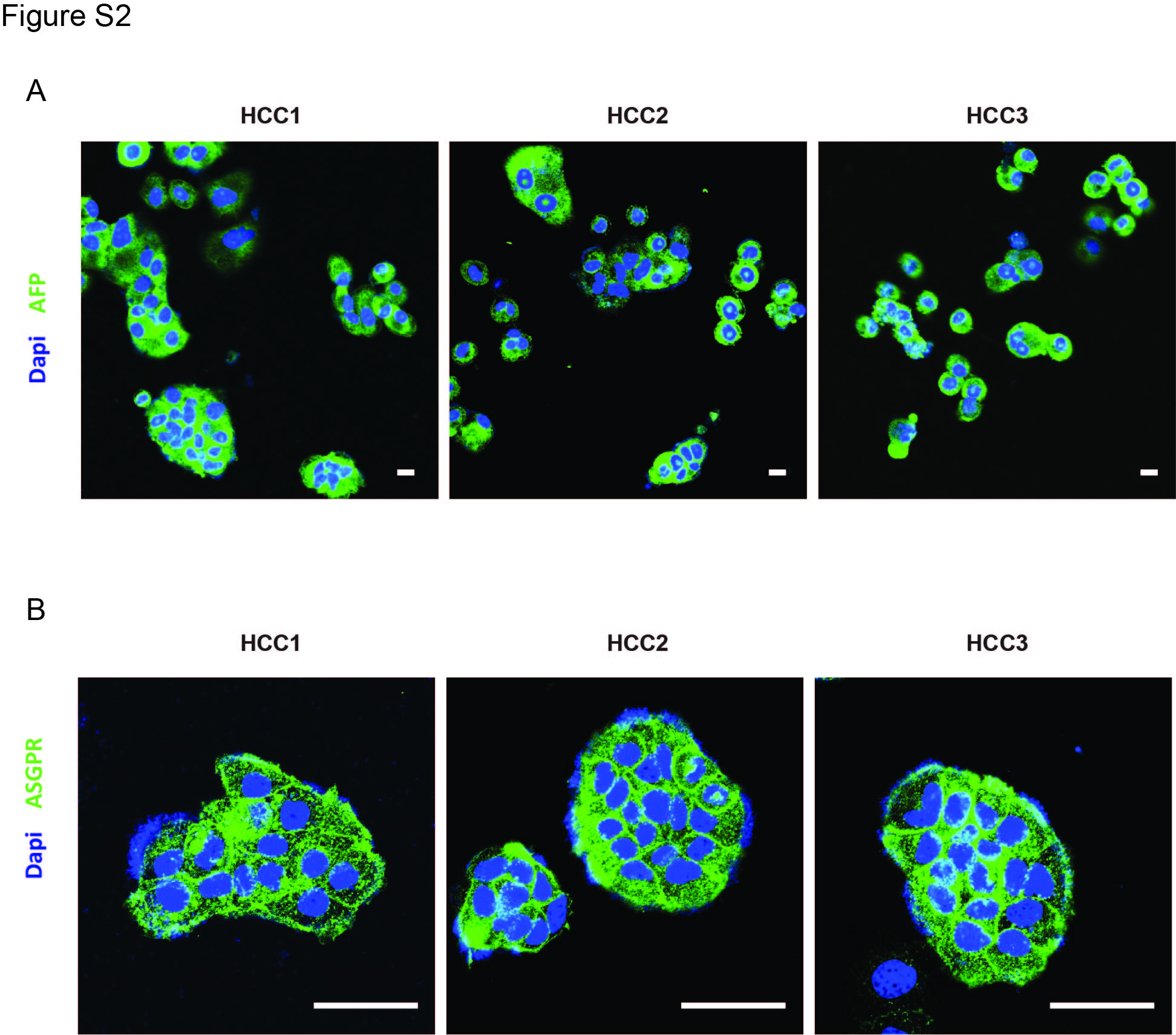

Supplement: Supplementary file 7 [file Image2.jpeg]

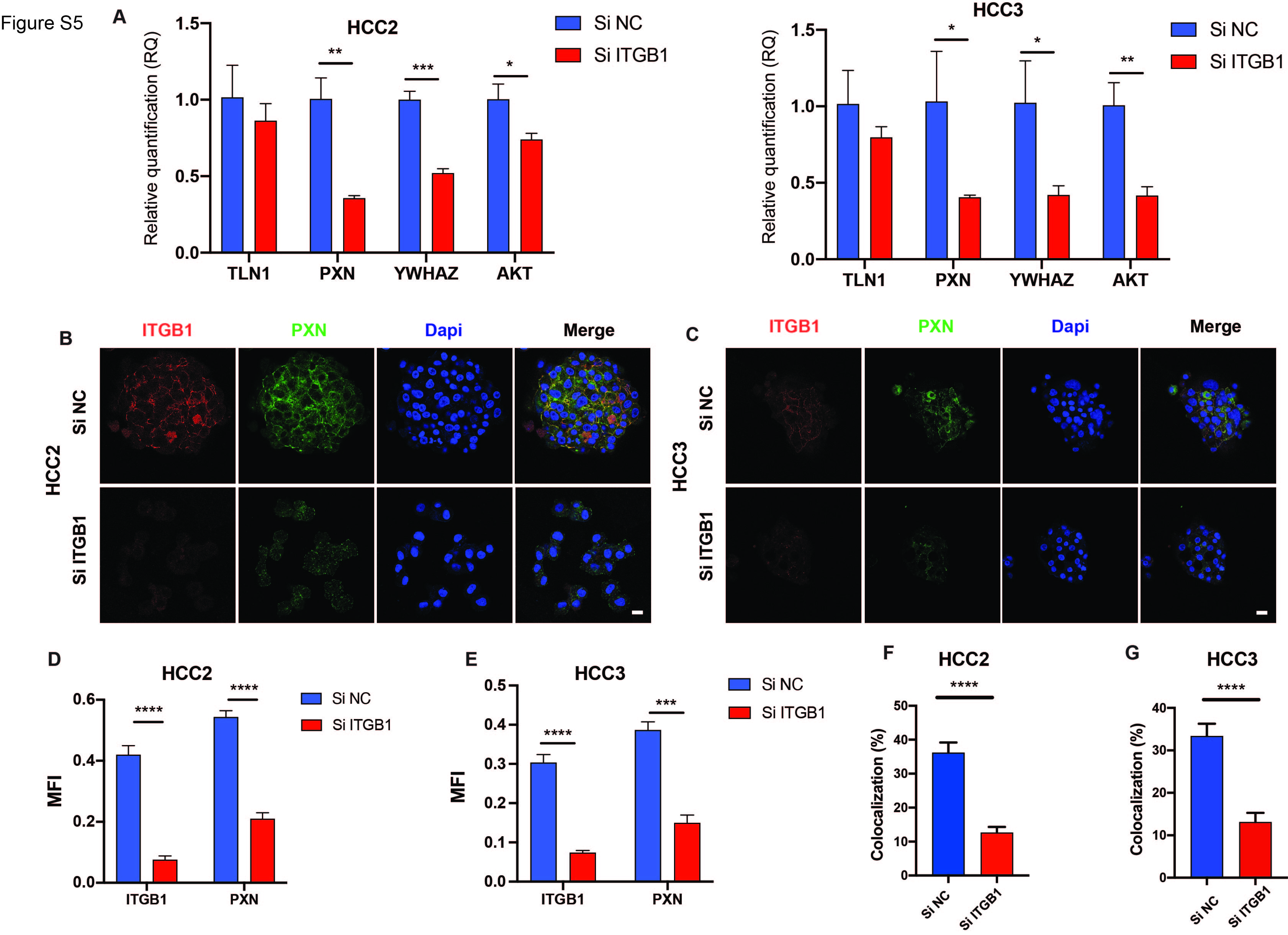

Supplement: Supplementary file 8 [file Image5.jpeg]

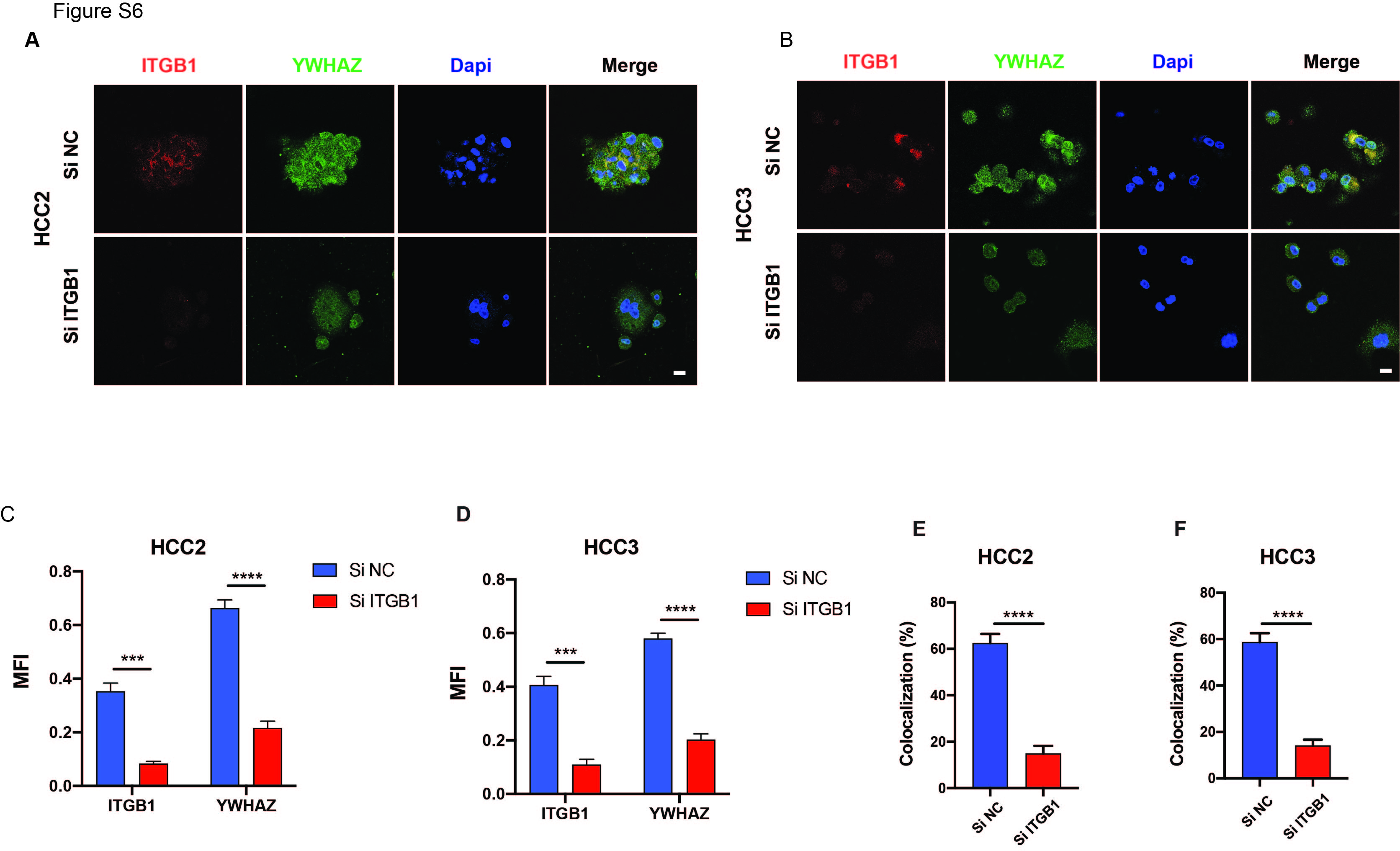

Supplement: Supplementary file 18 [file Image6.jpeg]
