# Supplementary material for: ITGB1 Drives Hepatocellular Carcinoma Progression by Modulating Cell Cycle Process Through PXN/YWHAZ/AKT Pathways
Source: Front Cell Dev Biol. 2021 Dec 17;9:711149. doi: 10.3389/fcell.2021.711149 (PMC8718767; doi:10.3389/fcell.2021.711149)

Applied Biosystems

GeneMapper ID v3.2

20210708

| Sample File | Sample Name | Panel | SQO | OS | SQ |
|---|---|---|---|---|---|
| 63_G08_CellLineAuthentication-2-0709.fsa | HCC31 | 21Plex_STR_Panel_v1.1 | | | |

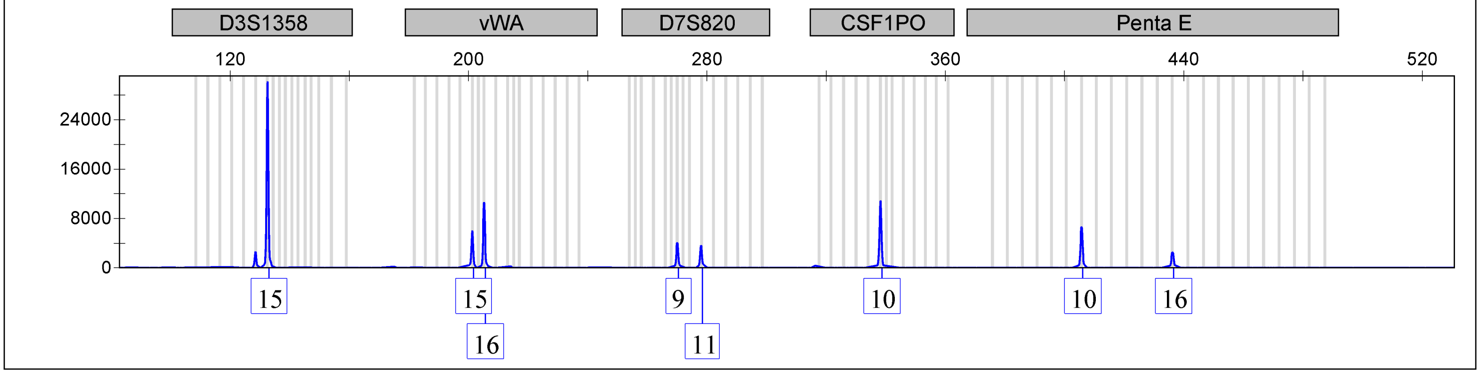


| | | | | | |
|---|---|---|---|---|---|
| 63_G08_CellLineAuthentication-2-0709.fsa | HCC31 | 21Plex_STR_Panel_v1.1 | | | |

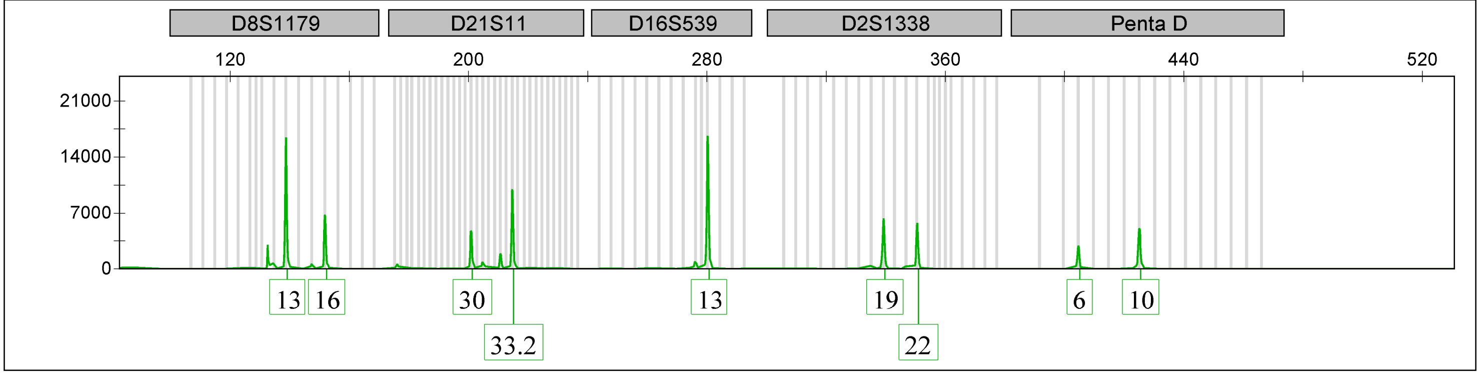


| | | | | | |
|---|---|---|---|---|---|
| 63_G08_CellLineAuthentication-2-0709.fsa | HCC31 | 21Plex_STR_Panel_v1.1 | | | |

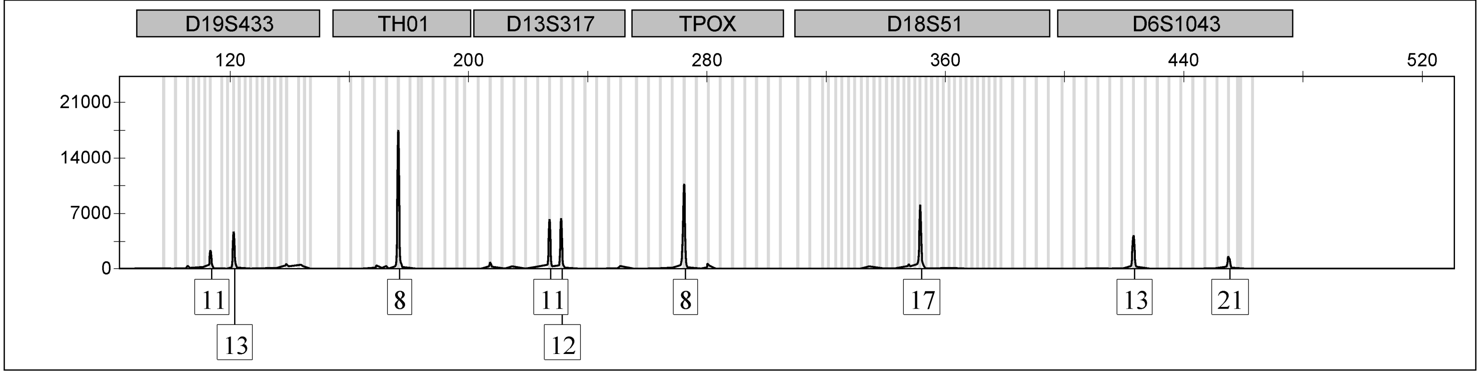


| | | | | | |
|---|---|---|---|---|---|
| 63_G08_CellLineAuthentication-2-0709.fsa | HCC31 | 21Plex_STR_Panel_v1.1 | | | |

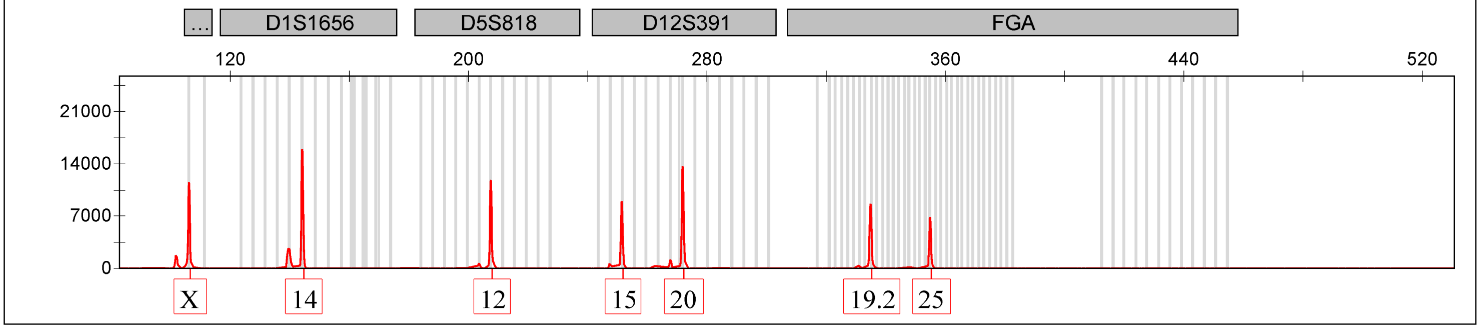

Supplement: Supplementary file 3 [file DataSheet4.PDF]
