# Supplementary material for: ITGB1 Drives Hepatocellular Carcinoma Progression by Modulating Cell Cycle Process Through PXN/YWHAZ/AKT Pathways
Source: Front Cell Dev Biol. 2021 Dec 17;9:711149. doi: 10.3389/fcell.2021.711149 (PMC8718767; doi:10.3389/fcell.2021.711149)

Sample File	Sample Name	Panel	SQO	OS	SQ
61_E08_CellLineAuthentication-2-0709.fsa	HCC23	21Plex_STR_Panel_v1.1			

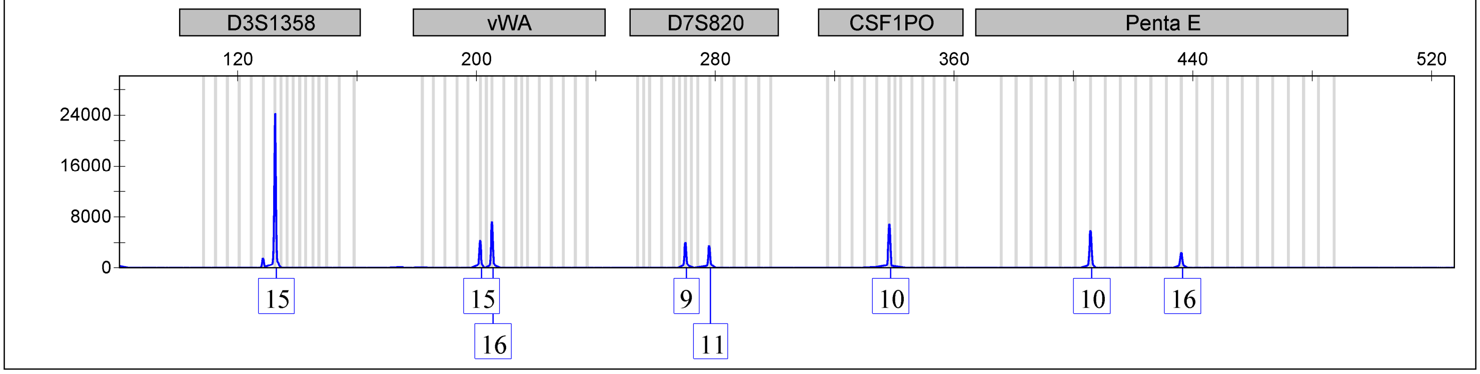

61_E08_CellLineAuthentication-2-0709.fsa	HCC23	21Plex_STR_Panel_v1.1			

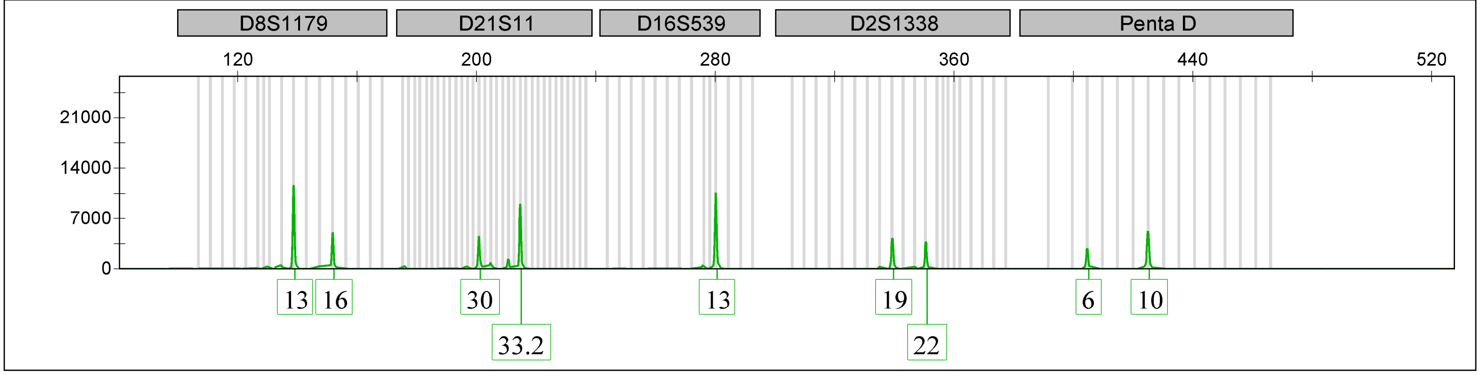

61_E08_CellLineAuthentication-2-0709.fsa	HCC23	21Plex_STR_Panel_v1.1			

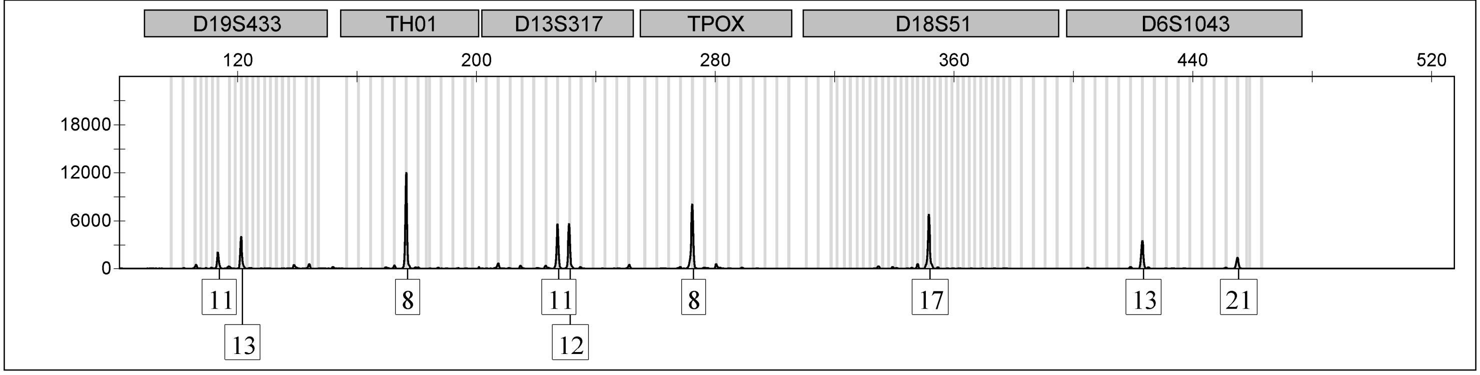

61_E08_CellLineAuthentication-2-0709.fsa	HCC23	21Plex_STR_Panel_v1.1			

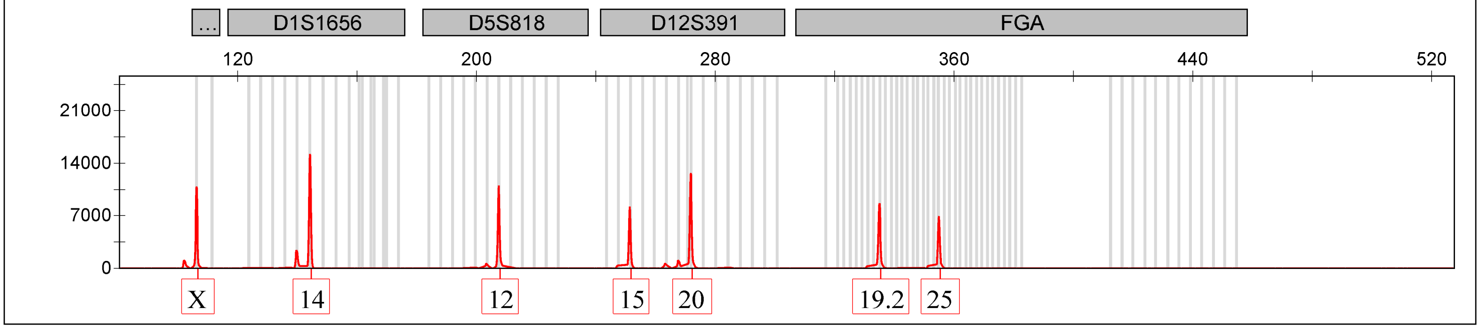

Supplement: Supplementary file 9 [file DataSheet3.PDF]
