# Supplementary material for: ITGB1 Drives Hepatocellular Carcinoma Progression by Modulating Cell Cycle Process Through PXN/YWHAZ/AKT Pathways
Source: Front Cell Dev Biol. 2021 Dec 17;9:711149. doi: 10.3389/fcell.2021.711149 (PMC8718767; doi:10.3389/fcell.2021.711149)
Supplement: Supplementary file 10 [file DataSheet1.PDF]

Table 1. Comparison of clinical characteristics between low ITGB1 group and high ITGB1 group in LIHC cohort

**Variable**	**ITGB1**		**Case NO.**	***P***
	**high**	**low**		
Age (mean±SD)	66.39±12.98	61.23±13.72		0.313
Gender				0.014
Male	116	133	249	
Female	69	51	120	
Pathologic stage				0.012
I/II	117	140	257	
III/IV	52	36	88	
NA	16	8	24	
Living status				0.197
Living	114	124	238	
Deceased	69	60	129	
