# Supplementary material for: ITGB1 Drives Hepatocellular Carcinoma Progression by Modulating Cell Cycle Process Through PXN/YWHAZ/AKT Pathways
Source: Front Cell Dev Biol. 2021 Dec 17;9:711149. doi: 10.3389/fcell.2021.711149 (PMC8718767; doi:10.3389/fcell.2021.711149)
Supplement: Supplementary file 11 [file Table5.DOCX]

| **EV** | **Cell No.** | **Cell name** | **Locus names** | | | | | | | | |
| --- | --- | --- | --- | --- | --- | --- | --- | --- | --- | --- | --- |
|  |  |  | **D5S818** | **D13S317** | **D7S820** | **D16S539** | **VWA** | **TH01** | **AM** | **TPOX** | **CSF1PO** |
|  | ***Query (Your Cell)*** | | **12,12** | **11,12** | **9,11** | **13,13** | **15,16** | **8,8** | **X,X** | **8,8** | **10,10** |

| 1.0(36/36) | **CRL-8024** | **PLC/PRF/5** | **['12', '12']** | **['11', '12']** | **['9', '11']** | **['13', '13']** | **['15', '16']** | **['8', '8']** | **['X', 'X']** | **['8', '8']** | **['10', '10']** |
| --- | --- | --- | --- | --- | --- | --- | --- | --- | --- | --- | --- |

| 1.0(36/36) | **CVCL_0485Worst** | **PLC/PRF/5** | **['12', '12']** | **['11', '12']** | **['9', '11']** | **['13', '13']** | **['15', '16']** | **['8', '8']** | **['X', 'X']** | **['8', '8']** | **['10', '10']** |
| --- | --- | --- | --- | --- | --- | --- | --- | --- | --- | --- | --- |

| 0.94(34/36) | **IFO50069** | **Alexander cells** | **['12', '12']** | **['11', '12']** | **['9', '11']** | **['13', '13']** | **['15', '16']** | **['7', '8']** | **['X', 'X']** | **['8', '8']** | **['10', '10']** |
| --- | --- | --- | --- | --- | --- | --- | --- | --- | --- | --- | --- |

| 0.94(34/36) | **JCRB0406** | **PLC/PRF/5** | **['12', '12']** | **['11', '12']** | **['9', '11']** | **['13', '13']** | **['15', '16']** | **['7', '8']** | **['X', 'X']** | **['8', '8']** | **['10', '10']** |
| --- | --- | --- | --- | --- | --- | --- | --- | --- | --- | --- | --- |

| 0.94(34/36) | **CVCL_0485Best** | **PLC/PRF/5** | **['12', '12']** | **['11', '12']** | **['9', '11']** | **['13', '13']** | **['15', '16']** | **['7', '8']** | **['X', 'X']** | **['8', '8']** | **['10', '10']** |
| --- | --- | --- | --- | --- | --- | --- | --- | --- | --- | --- | --- |

| 0.91(33/36) | **CVCL_0485 Worst** | **PLC/PRF/5** | **['12', '12']** | **['11', '12']** | **['9', '11']** | **['13', '13']** | **['15', '16']** | **['8', '8']** | **['']** | **['8', '8']** | **['10', '10']** |
| --- | --- | --- | --- | --- | --- | --- | --- | --- | --- | --- | --- |

| 0.86(31/36) | **CVCL_0485 Best** | **PLC/PRF/5** | **['12', '12']** | **['11', '12']** | **['9', '11']** | **['13', '13']** | **['15', '16']** | **['7', '8']** | **['']** | **['8', '8']** | **['10', '10']** |
| --- | --- | --- | --- | --- | --- | --- | --- | --- | --- | --- | --- |

| 0.78(28/36) | **CVCL_4016** | **SK-RC-45** | **['12', '12']** | **['11', '12']** | **['12', '12']** | **['13', '13']** | **['15', '16']** | **['6', '8']** | **['X', 'X']** | **['8', '8']** | **['10', '12']** |
| --- | --- | --- | --- | --- | --- | --- | --- | --- | --- | --- | --- |

| 0.72(26/36) | **CVCL_WR93** | **LaNCE hiPSC-43** | **['12', '12']** | **['11', '12']** | **['8', '11']** | **['11', '11']** | **['15', '16']** | **['9', '9']** | **['X', 'X']** | **['8', '8']** | **['10', '10']** |
| --- | --- | --- | --- | --- | --- | --- | --- | --- | --- | --- | --- |

| 0.72(26/36) | **CVCL_V001** | **NCI-H2869** | **['12', '13']** | **['8', '11']** | **['7', '8']** | **['13', '13']** | **['16', '16']** | **['8', '8']** | **['X', 'X']** | **['8', '8']** | **['10', '10']** |
| --- | --- | --- | --- | --- | --- | --- | --- | --- | --- | --- | --- |

| 0.67(24/36) | **568** | **NCI-H510A** | **['9', '12']** | **['11', '12']** | **['10', '11']** | **['13', '13']** | **['15', '15']** | **['7', '9.3']** | **['X', 'X']** | **['8', '8']** | **['10', '12']** |
| --- | --- | --- | --- | --- | --- | --- | --- | --- | --- | --- | --- |

| 0.67(24/36) | **CRL-1611** | **ACHN** | **['12', '12']** | **['12', '12']** | **['9', '11']** | **['12', '13']** | **['16', '17']** | **['8', '8']** | **['X', 'X']** | **['8', '11']** | **['11', '11']** |
| --- | --- | --- | --- | --- | --- | --- | --- | --- | --- | --- | --- |

| 0.67(24/36) | **HTB-184** | **NCI-H510A [H510A, NCI-H510]** | **['9', '12']** | **['11', '12']** | **['10', '11']** | **['13', '13']** | **['15', '15']** | **['7', '9.3']** | **['X', 'X']** | **['8', '8']** | **['10', '12']** |
| --- | --- | --- | --- | --- | --- | --- | --- | --- | --- | --- | --- |

| 0.67(24/36) | **HTB-54** | **Calu-1** | **['10', '12']** | **['11', '12']** | **['9', '10']** | **['11', '11']** | **['15', '16']** | **['9', '9.3']** | **['X', 'X']** | **['8', '8']** | **['10', '10']** |
| --- | --- | --- | --- | --- | --- | --- | --- | --- | --- | --- | --- |

| 0.67(24/36) | **RCB1962** | **ACHN** | **['12', '12']** | **['12', '12']** | **['9', '11']** | **['12', '13']** | **['16', '17']** | **['8', '8']** | **['X', 'X']** | **['8', '11']** | **['11', '11']** |
| --- | --- | --- | --- | --- | --- | --- | --- | --- | --- | --- | --- |

| 0.67(24/36) | **363** | **KYSE-70** | **['12', '12']** | **['11', '12']** | **['8', '11']** | **['13', '13']** | **['16', '18']** | **['7', '7']** | **['X', 'X']** | **['8', '8']** | **['12', '12']** |
| --- | --- | --- | --- | --- | --- | --- | --- | --- | --- | --- | --- |

| 0.67(24/36) | **JCRB0190** | **KYSE-70** | **['12', '12']** | **['11', '12']** | **['8', '11']** | **['13', '13']** | **['16', '18']** | **['7', '11']** | **['X', 'X']** | **['8', '8']** | **['12', '12']** |
| --- | --- | --- | --- | --- | --- | --- | --- | --- | --- | --- | --- |

| 0.67(24/36) | **752** | **1618-K** | **['12', '12']** | **['11', '11']** | **['7', '7']** | **['13', '13']** | **['16', '19']** | **['9', '9.3']** | **['X', 'X']** | **['8', '8']** | **['10', '10']** |
| --- | --- | --- | --- | --- | --- | --- | --- | --- | --- | --- | --- |

| 0.67(24/36) | **CVCL_A9W0** | **GSC#450** | **['11', '12']** | **['11', '13']** | **['9', '11']** | **['11', '13']** | **['17', '18']** | **['8', '9']** | **['X', 'X']** | **['8', '8']** | **['10', '10']** |
| --- | --- | --- | --- | --- | --- | --- | --- | --- | --- | --- | --- |

| 0.67(24/36) | **CVCL_6602** | **CHLA-20** | **['12', '15']** | **['11', '11']** | **['9', '11']** | **['13', '13']** | **['16', '17']** | **['6', '7']** | **['X', 'X']** | **['8', '8']** | **['10', '12']** |
| --- | --- | --- | --- | --- | --- | --- | --- | --- | --- | --- | --- |

| 0.67(24/36) | **CVCL_1356Worst** | **KYSE-70** | **['12', '12']** | **['11', '12']** | **['8', '11']** | **['13', '13']** | **['16', '18']** | **['7', '11']** | **['X', 'X']** | **['8', '8']** | **['12', '12']** |
| --- | --- | --- | --- | --- | --- | --- | --- | --- | --- | --- | --- |

| 0.67(24/36) | **CVCL_1356Best** | **KYSE-70** | **['12', '12']** | **['11', '12']** | **['8', '11']** | **['13', '13']** | **['16', '18']** | **['7', '7']** | **['X', 'X']** | **['8', '8']** | **['12', '12']** |
| --- | --- | --- | --- | --- | --- | --- | --- | --- | --- | --- | --- |

| 0.67(24/36) | **CVCL_Z066** | **TeloHAEC-GFP** | **['12', '12']** | **['9', '12']** | **['10', '11']** | **['12', '13']** | **['15', '16']** | **['6', '8']** | **['X', 'X']** | **['8', '8']** | **['11', '12']** |
| --- | --- | --- | --- | --- | --- | --- | --- | --- | --- | --- | --- |

| 0.67(24/36) | **CVCL_C615** | **FM92** | **['11', '12']** | **['11', '12']** | **['10', '11']** | **['13', '14']** | **['15', '19']** | **['8', '8']** | **['X', 'Y']** | **['8', '8']** | **['10', '11']** |
| --- | --- | --- | --- | --- | --- | --- | --- | --- | --- | --- | --- |

| 0.67(24/36) | **CVCL_0608Worst** | **Calu-1** | **['10', '12']** | **['11', '12']** | **['9', '9']** | **['11', '11']** | **['15', '16']** | **['9.3', '9.3']** | **['X', 'X']** | **['8', '8']** | **['10', '10']** |
| --- | --- | --- | --- | --- | --- | --- | --- | --- | --- | --- | --- |

| 0.67(24/36) | **CVCL_VP46** | **Mela14** | **['12', '12']** | **['11', '12']** | **['9', '11']** | **['10', '16']** | **['16', '20']** | **['9', '9']** | **['X', 'X']** | **['8', '8']** | **['10', '12']** |
| --- | --- | --- | --- | --- | --- | --- | --- | --- | --- | --- | --- |

| 0.67(24/36) | **CVCL_4Z31** | **TC-205** | **['12', '12']** | **['8', '10']** | **['10', '12']** | **['13', '13']** | **['15', '16']** | **['8', '9']** | **['X', 'X']** | **['8', '11']** | **['10', '10']** |
| --- | --- | --- | --- | --- | --- | --- | --- | --- | --- | --- | --- |

| 0.67(24/36) | **CVCL_1067Best** | **ACHN** | **['12', '12']** | **['12', '12']** | **['9', '11']** | **['12', '13']** | **['16', '17']** | **['8', '8']** | **['X', 'X']** | **['8', '11']** | **['11', '11']** |
| --- | --- | --- | --- | --- | --- | --- | --- | --- | --- | --- | --- |

| 0.67(24/36) | **CVCL_1067Worst** | **ACHN** | **['12', '12']** | **['12', '12']** | **['9', '11']** | **['12', '13']** | **['16', '17']** | **['8', '8']** | **['X', 'X']** | **['8', '11']** | **['11', '11']** |
| --- | --- | --- | --- | --- | --- | --- | --- | --- | --- | --- | --- |

| 0.67(24/36) | **CVCL_AQ27** | **CHLA-255** | **['12', '15']** | **['11', '11']** | **['9', '11']** | **['13', '13']** | **['16', '17']** | **['6', '7']** | **['X', 'X']** | **['8', '8']** | **['10', '12']** |
| --- | --- | --- | --- | --- | --- | --- | --- | --- | --- | --- | --- |

| 0.67(24/36) | **CVCL_S505** | **1618-K** | **['12', '12']** | **['11', '11']** | **['7', '7']** | **['13', '13']** | **['16', '19']** | **['9', '9.3']** | **['X', 'X']** | **['8', '8']** | **['10', '10']** |
| --- | --- | --- | --- | --- | --- | --- | --- | --- | --- | --- | --- |

| 0.67(24/36) | **CVCL_1565** | **NCI-H510A** | **['9', '12']** | **['11', '12']** | **['10', '11']** | **['13', '13']** | **['15', '15']** | **['7', '9.3']** | **['X', 'X']** | **['8', '8']** | **['10', '12']** |
| --- | --- | --- | --- | --- | --- | --- | --- | --- | --- | --- | --- |

| 0.67(24/36) | **CVCL_4036** | **UACC-1179** | **['12', '12']** | **['12', '12']** | **['8', '11']** | **['10', '13']** | **['16', '16']** | **['6', '6']** | **['X', 'X']** | **['8', '8']** | **['10', '10']** |
| --- | --- | --- | --- | --- | --- | --- | --- | --- | --- | --- | --- |

| 0.67(24/36) | **CVCL_0608Best** | **Calu-1** | **['10', '12']** | **['11', '12']** | **['9', '9']** | **['11', '11']** | **['15', '16']** | **['9', '9.3']** | **['X', 'X']** | **['8', '8']** | **['10', '10']** |
| --- | --- | --- | --- | --- | --- | --- | --- | --- | --- | --- | --- |

| 0.67(24/36) | **CVCL_Z065** | **TeloHAEC** | **['12', '12']** | **['9', '12']** | **['10', '11']** | **['12', '13']** | **['15', '16']** | **['6', '8']** | **['X', 'X']** | **['8', '8']** | **['11', '12']** |
| --- | --- | --- | --- | --- | --- | --- | --- | --- | --- | --- | --- |

| 0.61(22/36) | **CRL-1999** | **T/G HA-VSMC** | **['12', '13']** | **['11', '12']** | **['9', '10']** | **['12', '12']** | **['16', '16']** | **['7', '8']** | **['X', 'X']** | **['8', '8']** | **['10', '11']** |
| --- | --- | --- | --- | --- | --- | --- | --- | --- | --- | --- | --- |

| 0.61(22/36) | **359** | **DBTRG-05MG** | **['12', '13']** | **['9', '9']** | **['11', '11']** | **['12', '12']** | **['15', '16']** | **['8', '8']** | **['X', 'X']** | **['8', '8']** | **['10', '11']** |
| --- | --- | --- | --- | --- | --- | --- | --- | --- | --- | --- | --- |

| 0.61(22/36) | **377** | **TCC-SUP** | **['12', '12']** | **['11', '11']** | **['8', '9']** | **['9', '11']** | **['14', '16']** | **['6', '9.3']** | **['X', 'X']** | **['8', '8']** | **['10', '10']** |
| --- | --- | --- | --- | --- | --- | --- | --- | --- | --- | --- | --- |

| 0.61(22/36) | **461** | **ESS-1** | **['10', '12']** | **['8', '12']** | **['9', '11']** | **['12', '13']** | **['16', '17']** | **['7', '9']** | **['X', 'X']** | **['8', '8']** | **['10', '14']** |
| --- | --- | --- | --- | --- | --- | --- | --- | --- | --- | --- | --- |

| 0.61(22/36) | **CRL-1848** | **NCI-H292 [H292]** | **['13', '13']** | **['11', '12']** | **['10', '10']** | **['9', '13']** | **['16', '17']** | **['8', '8']** | **['X', 'X']** | **['8', '11']** | **['10', '10']** |
| --- | --- | --- | --- | --- | --- | --- | --- | --- | --- | --- | --- |
