# Supplementary material for: ITGB1 Drives Hepatocellular Carcinoma Progression by Modulating Cell Cycle Process Through PXN/YWHAZ/AKT Pathways
Source: Front Cell Dev Biol. 2021 Dec 17;9:711149. doi: 10.3389/fcell.2021.711149 (PMC8718767; doi:10.3389/fcell.2021.711149)
Supplement: Supplementary file 13 [file Table3.docx]

Table 1. Comparison of clinical characteristics between low ITGB1 group and high ITGB1 group in HCC cohort

| **Variable** | **ITGB1** | | **Case NO.** | ***P*** |
| --- | --- | --- | --- | --- |
|  | **high** | **low** |  |  |
| Age (mean±SD) | 66.39±12.98 | 61.23±13.72 |  | 0.313 |
| Gender | | |  | 0.014 |
| Male | 116 | 133 | 249 |  |
| Female | 69 | 51 | 120 |  |
| Pathologic stage | | |  | 0.841 |
| Ⅰ | 77 | 94 | 171 |  |
| Ⅱ | 40 | 46 | 86 |  |
| Ⅲ | 49 | 34 | 83 |  |
| Ⅳ | 3 | 2 | 5 |  |
| NA | 16 | 8 | 24 |  |
| Living status | | |  | 0.197 |
| Living | 114 | 124 | 238 |  |
| Deceased | 69 | 60 | 129 |  |
